# Estimation after subpopulation selection in adaptive seamless trials

**DOI:** 10.1002/sim.6506

**Published:** 2015-04-22

**Authors:** Peter K. Kimani, Susan Todd, Nigel Stallard

**Affiliations:** ^1^ Warwick Medical SchoolThe University of WarwickCoventryCV4 7ALU.K.; ^2^ Department of Mathematics and StatisticsThe University of ReadingRG6 6AXReadingU.K.

**Keywords:** adaptive seamless designs, phase II/III clinical trials, multi‐arm multi‐stage trials, subpopulation, subgroup analysis

## Abstract

During the development of new therapies, it is not uncommon to test whether a new treatment works better than the existing treatment for all patients who suffer from a condition (full population) or for a subset of the full population (subpopulation). One approach that may be used for this objective is to have two separate trials, where in the first trial, data are collected to determine if the new treatment benefits the full population or the subpopulation. The second trial is a confirmatory trial to test the new treatment in the population selected in the first trial. In this paper, we consider the more efficient two‐stage adaptive seamless designs (ASDs), where in stage 1, data are collected to select the population to test in stage 2. In stage 2, additional data are collected to perform confirmatory analysis for the selected population. Unlike the approach that uses two separate trials, for ASDs, stage 1 data are also used in the confirmatory analysis. Although ASDs are efficient, using stage 1 data both for selection and confirmatory analysis introduces selection bias and consequently statistical challenges in making inference. We will focus on point estimation for such trials. In this paper, we describe the extent of bias for estimators that ignore multiple hypotheses and selecting the population that is most likely to give positive trial results based on observed stage 1 data. We then derive conditionally unbiased estimators and examine their mean squared errors for different scenarios.©2015 The Authors. *Statistics in Medicine* Published by JohnWiley & Sons Ltd.

## 1 Introduction

In drug development, it is not uncommon to have a hypothesis selection stage followed by a confirmatory analysis stage. In the hypothesis selection stage, data are collected to test multiple hypotheses, with the hypothesis that is most likely to give positive trial results selected to be tested in the confirmatory analysis stage. In this paper, we will consider two‐stage adaptive seamless designs (ASDs) in which the hypothesis selection stage (stage 1) and the confirmatory analysis stage (stage 2) are two parts of a single trial, with hypothesis selection performed at an interim analysis. An alternative to an ASD is to have two separate trials, separate in the sense that stage 1 data are only used for hypothesis selection and the confirmatory analysis uses stage 2 data only. However, an ASD is more efficient than having two separate trials because, as data from both stages of an adaptive seamless trial are used in the final confirmatory analysis, for the same power, fewer patients would be required in stage 2 of an adaptive seamless trial than in the setting with two separate trials hence saving resources. The two‐stage adaptive seamless trial can also be designed so that it is more efficient than having a trial with a single stage, where a single analysis is used to select and test the best hypothesis, for example using the Bonferroni test or the Dunnett test 1.

Much work has been undertaken on ASDs where the multiple hypotheses arise as a result of comparing a control to several experimental treatments in stage 1. Based on stage 1 data, the most promising experimental treatment is selected to continue to stage 2 together with the control. We refer to this as treatment selection. In stage 1, available patients are randomly allocated to the control and all the experimental treatments while in stage 2, patients are randomly allocated to the control and the most promising experimental treatment. The experimental treatments may be distinct treatments or different doses of a single experimental treatment. Treatment selection in ASDs is described in more detail in 2, 3, 4, 5, 6, 7, 8, 9, 10 among others. A challenge with such adaptive seamless trials is that selecting the most promising experimental treatment in stage 1 introduces selection bias because the superiority of the selected experimental treatment may be by chance. Consequently, appropriate confirmatory analysis needs to account for using biased stage 1 data. Hypothesis testing methods that control type I error rate have been developed or described in 2, 3, 4, 5, 6, 7, 8. Point estimators that adjust for treatment selection have been developed in 11, 12, 13, 14, 15 while confidence intervals that adjust for treatment selection have been considered in 7, 13, 16, 17, 18, 19.

In this paper, we consider the case where multiple hypotheses arise because in stage 1, a control is compared with a single experimental treatment in several subpopulations. Based on stage 1 data, the subpopulation in which the experimental treatment shows most benefit over the control is selected to be tested further in stage 2. We refer to this as subpopulation selection. In stage 1, patients are recruited from all subpopulations while in stage 2, patients are recruited from the selected subpopulation only and randomly allocated to the control and the experimental treatment. Subpopulation (subgroup) analysis has been considered in many trials, encompassing many disease areas such as Alzheimer's 20, epilepsy 21 and cancer 22. Most of these trials are single stage but investigators are beginning to design two‐stage adaptive seamless trials for subpopulation selection such as the trial described in 23. The subpopulation may be defined based on baseline disease severity 20, 21, age group 24 or a genetic biomarker 22 among other criteria. As in 23, we will assume that the subpopulations are pre‐specified. The case of subpopulation selection in ASDs is described in more detail in 23, 25, 26, 27, 28.

As in the case of treatment selection, subpopulation selection introduces selection bias because the most promising subpopulation is selected to be tested in stage 2. Methods for hypothesis testing in two‐stage adaptive seamless trials with subpopulation selection that control type I error rate have been developed 5, 23, 26. Some of these methods were initially developed for hypothesis testing following treatment selection. It has been possible to test hypotheses after subpopulation selection using some hypothesis testing methods developed for treatment selection because these methods are not fully parametric. For example, Brannath *et al*. 23 have shown that the method described in 5, 8 can be used for hypothesis testing in the case of subpopulation selection. Estimation after adaptive seamless trials with subpopulation selection has not been considered. However, for confidence intervals, it is possible to use the duality between hypothesis testing and confidence intervals as described for the case of treatment selection in 7, 18, 19. For point estimation, the methods proposed for treatment selection 11, 12, 13, 14, 15 are based on explicit distributions and so their extension for use in subpopulation selection testing is not straightforward.

In this paper, we will consider point estimation after two‐stage ASDs where stage 1 data are used to perform subpopulation selection. Spiessens and Debois 25 have described the possible scenarios for subgroup analysis based on how the subpopulations are nested within each other and about which subpopulations the investigators want to draw inference. We will consider the scenario where the effect is considered in the full population and in a single subpopulation. This scenario seems to be of most practical importance having been considered in methodological work related to actual trial designs 23, 26. In the discussion, we will describe how the estimators we develop can be extended to some of the other scenarios in 25.

We organise the rest of the paper as follows. In Section 2, we first describe the setting of interest while introducing notation and then define the naive estimator, which ignores subpopulation selection before deriving a conditionally unbiased estimator. Section 3 gives an example that is used to demonstrate how to compute the naive and unbiased estimators and compare the two estimators for specific cases. We assess the mean squared error of the unbiased estimator in relation to the naive estimator in Section 4. The findings in the paper are discussed in Section 5.

## 2 Estimation in adaptive seamless designs for subpopulation selection

### 2.1 Setting and notation

**Figure 1:**
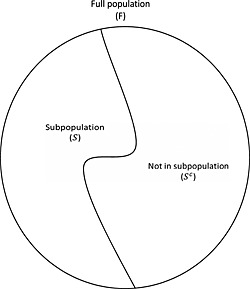
Partitioning of the full population.

As described in Section 1, we will consider an ASD in which a control is compared with an experimental treatment in a population of patients that consists of a subpopulation that may benefit from the experimental treatment more than the full population. In stage 1, patients are recruited from the full population but it is expected that a subpopulation may benefit more so that the focus at the end of the trial may be in the subpopulation only. Figure 1 shows how the patients in stage 1 are partitioned. The subpopulation, defined by some characteristics such as a biomarker and which we refer to as *S*, is part of the full population. We refer to the full population as *F* and the part of *F* that is not part of *S* as *S* ^*c*^. We assume *S* comprises a proportion *p*_*S*_ of *F*. At first, we focus on the case of known *p*_*S*_ before considering the case of unknown *p*_*S*_ in Section 2.4. We will use subscripts *S*, *S* ^*c*^ and *F* to indicate notation that corresponds to populations *S*, *S* ^*c*^ and *F*, respectively. The patients are randomised to the control treatment and the experimental treatment. We assume randomisation is stratified such that in each of *S* and *S* ^*c*^, the number of patients randomised to the control is equal to the number of patients randomised to the experimental treatment. Based on stage 1 data, the trial continues to stage 2 either with *F* or with *S*.

We assume outcomes from patients are normally distributed with unknown means and known common variance *σ* ^2^. We are interested in the unknown treatment difference between means for the control and the experimental treatment. Table 1 shows the key notation that we will use in this paper. We denote the unknown true treatment differences in *S* and *S* ^*c*^ by *θ*_*S*_ and $\theta_{S^{c}}$, respectively. We denote stage 1 sample mean differences for *S* and *S* ^*c*^ by *X* and *Y*, respectively and the stage 1 sample mean difference for *F* by *Z*, which can be expressed by $Z=p_{S}X+p_{S^{c}}Y$, where $p_{S^{c}}=1-p_{S}$. We assume that a total of *n*_1_ patients are recruited in stage 1 so that *S*_*X*_=*p*_*S*_ *n*_1_ patients are from *S* with *S*_*X*_/2 randomly allocated each to the control and the experimental treatment. The remaining (*n*_1_−*S*_*X*_) patients are from *S* ^*c*^ with (*n*_1_−*S*_*X*_)/2 randomly allocated each to the control and the experimental treatment. Note that $X∼N\left(\theta_{S},\sigma_{X}^{2}\right)$, where $\sigma_{X}^{2}=4\sigma^{2}/S_{X}$ and $Y∼N\left(\theta_{S^{c}},\sigma_{Y}^{2}\right)$, where $\sigma_{Y}^{2}=4\sigma^{2}/\left(n_{1}-S_{X}\right)$.

**Table 1:** Summary of notation.

			Stage 1		Stage 2		Stages 1 and 2		
Selected	Sub‐	True parameter	Sample	Variance of	Sample	Variance of	Naive	Sufficient	Unbiased
population	population	value	mean	sample mean	mean	sample mean	estimator	statistic	estimator
*S*	*S*	*θ*_*S*_	*X*	$\sigma_{X}^{2}$	*U*	$\tau_{U}^{2}$	*D*_*S*,*N*_	*Z*_*S*_	*D*_*S*,*U*_
	*S* ^*c*^	$\theta_{S^{c}}$	*Y*	$\sigma_{Y}^{2}$	—	—	—	—	—
*F*	*S*	*θ*_*S*_	*X*	$\sigma_{X}^{2}$	*V*	$\tau_{V}^{2}$	$D_{S,N}^{F}$	$Z_{S}^{F}$	$D_{S,U}^{F}$
	*S* ^*c*^	$\theta_{S^{c}}$	*Y*	$\sigma_{Y}^{2}$	*W*	$\tau_{W}^{2}$	$D_{S^{c},N}^{F}$	$Z_{S^{c}}$	$D_{S^{c},U}^{F}$
	*S* + *S* ^*c*^	*θ*_*F*_	*Z*	—	—	—	*D*_*F*,*N*_	—	*D*_*F*,*U*_

The observed values for *X*, *Y* and *Z* are denoted by *x*, *y* and *z*, respectively. The trial continues to stage 2 with *S* if *x* > (*z* + *b*), which is equivalent to *x* > *y* + *b*/(1 − *p*_*S*_), where *b* is a number chosen such that the trial continues with *S* if the effect of the new treatment is sufficiently larger in *S* than in *F*. The trial continues to stage 2 with *F* if x≤(z+b), which is equivalent to $x\leq y+b/\left(1-p_{S}\right)$. In stage 2, a total of *n*_2_ patients are recruited. If *S* is selected, all the *n*_2_ patients will be from *S* with *n*_2_/2 patients randomly allocated each to the control and the experimental treatment. If *F* is selected, *S*_*V*_=*p*_*S*_ *n*_2_ patients will be from *S* and (*n*_2_−*S*_*V*_) patients will be from *S* ^*c*^.

If *S* is selected to continue to stage 2, the objective is to estimate *θ*_*S*_ while if *F* is selected to continue to stage 2, the objective is to estimate $\theta_{F}=p_{S}\theta_{S}+p_{S^{c}}\theta_{S^{c}}$. Therefore, the parameter of interest at the end of the two‐stage trial, which we denote by *θ*, is random and is defined by

(1) $$\theta =\left\{\begin{array}{l} \theta_{S}\;\;\;\;\;\text{if}S\text{is selected}\\ \theta_{F}\;\;\;\;\text{if}F\text{is selected}. \end{array}\right.$$

We will consider two estimators for *θ*, namely, the naive and the unbiased estimators. As shown in Table 1, when *S* is selected, we denote the naive estimator for *θ*_*S*_ by *D*_*S*,*N*_. When *F* is selected, we denote the naive estimators for *θ*_*S*_, $\theta_{S^{c}}$ and *θ*_*F*_ by $D_{F,N}^{F}$, $D_{S^{c},N}^{F}$ and *D*_*F*,*N*_, respectively. We define the naive estimator for *θ* as

(2) $$D_{N}=\left\{\begin{array}{l} D_{S,N}\;\;\;\;\;\text{if}S\text{is selected}\\ D_{F,N}\;\;\;\;\;\text{if}F\text{is selected}. \end{array}\right.$$

We give the expressions for the naive estimators *D*_*S*,*N*_, $D_{S,N}^{F}$, $D_{S^{c},N}^{F}$ and *D*_*F*,*N*_ and derive their bias functions in Section 2.2. In the following, we derive uniformly minimum variance unbiased estimators (UMVUEs) for *θ*_*S*_ and $\theta_{S^{c}}$. As indicated in Table 1, when *S* is selected, we denote the UMVUE for *θ*_*S*_ by *D*_*S*,*U*_. When *F* is selected, we denote the UMVUEs for *θ*_*S*_ and $\theta_{S^{c}}$ by $D_{S,U}^{F}$ and $D_{S^{c},U}^{F}$, respectively. Note that $D_{\text{F, U}}=p_{S}D_{S,U}^{F}+p_{S^{c}}D_{S^{c},U}^{F}$ is an unbiased estimator for *θ*_*F*_. We define the unbiased estimator for *θ* as

(3) $$D_{U}=\left\{\begin{array}{l} D_{S,U}\;\;\;\;\;\text{if}S\text{is selected}\\ D_{F,U}\;\;\;\;\;\text{if}F\text{is selected}. \end{array}\right.$$

We derive the expressions for UMVUEs *D*_*S*,*U*_, $D_{S,U}^{F}$ and $D_{S^{c},U}^{F}$ in Section 2.3.

We will compare the naive (*D*_*N*_) and the unbiased (*D*_*U*_) estimators for *θ* by evaluating the bias for *D*_*N*_ and the mean squared errors (MSEs) for *D*_*N*_ and *D*_*U*_. We will evaluate biases and MSEs conditional on the selection made and so for the naive estimator, we will derive expressions for biases for *D*_*S*,*N*_ and *D*_*F*,*N*_ separately. Similarly, the MSEs for *D*_*N*_ and *D*_*U*_ will be evaluated conditional on the selection made. Note that if an estimator is unbiased conditional on selection, it is also unconditionally unbiased.

### 2.2 The naive estimator

In this section, we describe the naive estimator for *θ* defined by equation (2) and derive simple expressions for its bias function. When *S* is selected, a possible naive estimator for *θ*_*S*_, which we denote by *D*_*S*,*N*_ in expression (2), is the two‐stage sample mean given by

(4) $$D_{S,N}=t_{S}X+\left(1-t_{S}\right)U,$$

where *U* denotes the stage 2 sample mean for patients in *S* and *t*_*S*_=*S*_*X*_/(*S*_*X*_+*n*_2_) is the proportion of patients in *S* who are in stage 1. The expected value for *D*_*S*,*N*_ can be expressed as *E*(*D*_*S*,*N*_) = *t*_*S*_ *E*(*X*|*X* > *Y* ^*^) + (1 − *t*_*S*_)*θ*_*S*_, where *Y* ^*^=*Y* + *b*/(1 − *p*_*S*_) so that the bias for *D*_*S*,*N*_ is given by

(5) $$\text{Bias}\left(D_{S,N}\right)=E\left(D_{S,N}\right)-\theta_{S}=t_{S}\left\{E\;\left(X|X>Y^{*}\right)-\theta_{S}\right\}=t_{S}\left\{\frac{E\left(X1_{\left[X>Y^{*}\right]}\right)}{\mathrm{Pr}\left(X>Y^{*}\right)}-\theta_{S}\right\},$$

where $1_{\left[X>Y^{*}\right]}$ denotes the indicator function for *X* > *Y* ^*^. Following the last expression in Appendix C.1 in 15, Pr(*X* > *Y* ^*^) can be expressed as follows

(6) $$\mathrm{Pr}\left(X>Y^{*}\right)=\overset{\infty }{\underset{-\infty }{\int }}\frac{1}{\sigma_{X}}\varphi \left(\frac{t-\theta_{S}}{\sigma_{X}}\right)\Phi \left(\frac{t-\theta_{S^{c}}^{*}}{\sigma_{Y}}\right)\mathrm{dt},$$

where *σ*_*X*_ and *σ*_*Y*_ are as defined in Section 2.1, $\theta_{S^{c}}^{*}=\theta_{S^{c}}+b/\left(1-p_{S}\right)$ and *φ* and Φ denote the density and distribution functions of the standard normal, respectively. Also, following Appendix C.1 in 15, $E\;\left(X1_{\left[X>Y^{*}\right]}\right)$ can be expressed as

(7) $$E\;\left(X1_{\left[X>Y^{*}\right]}\right)=\overset{\infty }{\underset{-\infty }{\int }}\frac{t}{\sigma_{X}}\varphi \left(\frac{t-\theta_{S}}{\sigma_{X}}\right)\Phi \left(\frac{t-\theta_{S^{c}}^{*}}{\sigma_{Y}}\right)\mathrm{dt}.$$

The expressions for Pr(*X* > *Y* ^*^)and $E\;\left(X1_{\left[X>Y^{*}\right]}\right)$ are substituted in expression (5) to obtain the bias function for *D*_*S*,*N*_.

If *F* is selected to continue to stage 2, we are seeking an estimator for *θ*_*F*_. Let $t_{S}^{F}=S_{X}/(S_{X}+S_{V})$ and $t_{S^{c}}^{F}=\left(n_{1}-S_{X}\right)/(n_{1}+n_{2}-S_{X}-S_{V})$ denote the proportion of patients recruited in stage 1 from *S* and *S* ^*c*^, respectively, and as indicated in Table 1, let *V* and *W* denote the stage 2 sample means for *S* and *S* ^*c*^, respectively. If *F* is selected to continue to stage 2, possible naive estimators for *θ*_*S*_ and $\theta_{S^{c}}$, which we denote by $D_{S,N}^{F}$ and $D_{S^{c},N}^{F}$, respectively in Section 2.1, are the two‐stage sample means $D_{S,N}^{F}=t_{S}^{F}X+\left(1-t_{S}^{F}\right)V$ and $D_{S^{c},N}^{F}=t_{S^{c}}^{F}Y+\left(1-t_{S^{c}}^{F}\right)W$. Consequently, a naive estimator for *θ*_*F*_, which we denote by *D*_*F*,*N*_ in expression (2), could be

(8) $$D_{F,N}=p_{S}D_{S,N}^{F}+p_{S^{c}}D_{S^{c},N}^{F}.$$

The bias for *D*_*F*,*N*_ can be expressed as

(9) $$\text{Bias}(D_{F,N})=p_{S}\cdot \text{Bias}\;\left(D_{S,N}^{F}\right)+p_{S^{c}}\cdot \text{Bias}\;\left(D_{S^{c},N}^{F}\right),$$

where $\text{Bias}\;\left(D_{S,N}^{F}\right)=E\;\left(D_{S,N}^{F}\right)-\theta_{S}$ and $\text{Bias}\;\left(D_{S^{c},N}^{F}\right)=E\;\left(D_{S^{c},N}^{F}\right)-\theta_{S^{c}}$ are given by

(10) $$\text{Bias}\;\left(D_{S,N}^{F}\right)=t_{S}^{F}\left\{\frac{E\;\left(X1_{\left[X\leq Y^{*}\right]}\right)}{\mathrm{Pr}\left(X\leq Y^{*}\right)}-\theta_{S}\right\}\;\;\;\text{and}\;\;\;\text{Bias}\;\left(D_{S^{c},N}^{F}\right)=t_{S^{c}}^{F}\left\{\frac{E\;\left(Y1_{\left[X\leq Y^{*}\right]}\right)}{\mathrm{Pr}\left(X\leq Y^{*}\right)}-\theta_{S^{c}}\right\},$$

where $1_{\left[X\leq Y^{*}\right]}$ denotes the indicator function for $X\leq Y^{*}$. As for the expressions for Pr(*X* > *Y* ^*^) and $E\;\left(X1_{\left[X>Y^{*}\right]}\right)$, $\mathrm{Pr}\left(X\leq Y^{*}\right)$ and $E\;\left(Y1_{\left[X\leq Y^{*}\right]}\right)$ in the earlier expressions can be respectively expressed as

$$\mathrm{Pr}\left(X\leq Y^{*}\right)=\overset{\infty }{\underset{-\infty }{\int }}\frac{1}{\sigma_{Y}}\varphi \left(\frac{t-\theta_{S^{c}}}{\sigma_{Y}}\right)\Phi \left(\frac{t-\theta_{S}^{*}}{\sigma_{X}}\right)\mathrm{dt},$$

where $\theta_{S}^{*}=\theta_{S}-b/\left(1-p_{S}\right)$ and

$$E\;\left(Y1_{\left[X\leq Y^{*}\right]}\right)=\overset{\infty }{\underset{-\infty }{\int }}\frac{t}{\sigma_{Y}}\varphi \left(\frac{t-\theta_{S^{c}}}{\sigma_{Y}}\right)\Phi \left(\frac{t-\theta_{S}^{*}}{\sigma_{X}}\right)\mathrm{dt}.$$

For the case we consider here where the population has two partitions, a simple expression for $E\;\left(X1_{\left[X\leq Y^{*}\right]}\right)$ is $\theta_{S}-E\;\left(X1_{\left[X>Y^{*}\right]}\right)$. Appendix C.2 in 15 has expressions with a single integral that can be modified when the partitioning of the population is more complex.

The aforementioned expressions for $\mathrm{Pr}\left(X\leq Y^{*}\right)$, $E\;\left(Y1_{\left[X\leq Y^{*}\right]}\right)$ and $E\;\left(X1_{\left[X\leq Y^{*}\right]}\right)$ are used to obtain the bias functions for $D_{S,N}^{F}$, $D_{S^{c},N}$ and *D*_*F*,*N*_. We will use the bias functions for *D*_*S*,*N*_, $D_{S,N}^{F}$, $D_{S^{c},N}$ and *D*_*F*,*N*_ that we have derived in this section to show the extent of the bias for the naive estimator in Section 4.1, which necessitates the need for an unbiased estimator for *θ* such as the one we derive in the following section.

### 2.3 Conditionally unbiased estimator for *θ* when the prevalence of the subpopulation is known

In this section, we derive an estimator for *θ* that is unbiased conditional on the selection made. To do this, we need the densities of the stage 2 means. The notation for the variances for the stage 2 sample means is given in Table 1. If *S* is selected to continue to stage 2, *U* is normally distributed with variance $\tau_{U}^{2}=4\sigma^{2}/n_{2}$. If *F* is selected to continue to stage 2, *V* and *W* are normally distributed with variances $\tau_{V}^{2}=4\sigma^{2}/S_{V}$ and $\tau_{W}^{2}=4\sigma^{2}/(n_{2}-S_{V})$, respectively. Also, to derive the unbiased estimators, we need sufficient statistics and these will be vectors that include the weighted sums of stages 1 and 2 means. The notation for the weighted means for the two alternative choices of population is given in the second last column in Table 1.

To obtain the unbiased estimator, we use the Rao–Blackwell theorem (for example, 29). This states that, to obtain the UMVUE for a parameter, one identifies an unbiased estimator for the parameter of interest and then derives its expectation conditional on a complete and sufficient statistic. Let *Q*_*S*_ denote the event *X* > *Y* + *b*/(1 − *p*_*S*_). Conditional on *Q*_*S*_, *U* is an unbiased estimator for *θ*_*S*_ so that if we can identify a sufficient and complete statistic for estimating *θ*_*S*_, we can use the Rao–Blackwell theorem to derive the UMVUE for *θ*_*S*_. Define *Z*_*S*_=(*τ*_*U*_/*σ*_*X*_)*X* + (*σ*_*X*_/*τ*_*U*_)*U*. We describe in Appendix A that conditional on *Q*_*S*_, (*Y*,*Z*_*S*_) is the sufficient and complete statistic for *θ*_*S*_ and that the UMVUE for *θ*_*S*_, *E*[*U*|*Y*,*Z*_*S*_,*Q*_*S*_], which we denote by *D*_*S*,*U*_ in Section 2.1, is given by

(11) $$D_{S,U}=D_{S,N}-\frac{\tau_{U}^{2}}{\sqrt{\sigma_{X}^{2}+\tau_{U}^{2}}}\frac{\varphi \{\;f_{U}(X,Y)\}}{\Phi \{\;f_{U}(X,Y)\}},$$

where, after substituting *p*_*S*_ with *S*_*X*_/*n*_1_ in the expression for *f*_*U*_(*x*,*y*) given in Appendix A,

$$f_{U}(X,Y)=\left(\frac{\sqrt{\sigma_{X}^{2}+\tau_{U}^{2}}}{\sigma_{X}^{2}}\right)\left\{D_{S,N}-\left(Y+\frac{b}{1-S_{X}/n_{1}}\right)\right\}.$$

We have substituted *p*_*S*_ with *S*_*X*_/*n*_1_ in the expression for *f*_*U*_(*X*,*Y*) and also in the expressions for *f*_*V*_(*X*,*Y*) and *f*_*W*_(*X*,*Y*) defined in the following so that estimators in this section and corresponding estimators in Section 2.4 have the same expressions.

Let *Q*_*F*_ denote the event $X\leq Y+b/\left(1-p_{S}\right)$. Conditional on *Q*_*F*_, *V* and *W* are unbiased estimators for *θ*_*S*_ and $\theta_{S^{c}}$, respectively so that if appropriate sufficient and complete statistics for *θ*_*S*_ and $\theta_{S^{c}}$ can be identified, the UMVUEs for *θ*_*S*_ and $\theta_{S^{c}}$ can be obtained using the Rao–Blackwell theorem. Define $Z_{S}^{F}=\left(\tau_{V}/\sigma_{X}\right)X+\left(\sigma_{X}/\tau_{V}\right)V$ and $Z_{S^{c}}^{F}=\left(\tau_{W}/\sigma_{Y}\right)Y+\left(\sigma_{Y}/\tau_{W}\right)W$. We show in Appendix B that conditional on *Q*_*F*_, $\left(\;Y,Z_{S}^{F}\right)$ and $\left(X,Z_{S^{c}}^{F}\right)$ are sufficient and complete statistics for *θ*_*S*_ and $\theta_{S^{c}}$, respectively and that the UMVUE for *θ*_*S*_, $E\left[V|Y,Z_{S}^{F},Q_{F}\right]$, which we denote by $D_{S,U}^{F}$ in Section 2.1, is given by

(12) $$D_{S,U}^{F}=D_{S,N}^{F}+\frac{\tau_{V}^{2}}{\sqrt{\sigma_{X}^{2}+\tau_{V}^{2}}}\frac{\varphi \left\{\;f_{V}(X,Y)\right\}}{\Phi \left\{\;f_{V}(X,Y)\right\}},$$

where, after substituting *p*_*S*_ with *S*_*X*_/*n*_1_ in the expression for *f*_*V*_(*x*,*y*) given in Appendix B,

$$f_{V}(X,Y)=\left(\frac{\sqrt{\sigma_{X}^{2}+\tau_{V}^{2}}}{\sigma_{X}^{2}}\right)\left\{\left(Y+\frac{b}{1-S_{X}/n_{1}}\right)-D_{S,N}^{F}\right\}$$

and that the UMVUE for $\theta_{S^{c}},E\left[W|X,Z_{S^{c}}^{F},Q_{F}\right]$, which we denote by $D_{S^{c},U}^{F}$ in Section 2.1, is given by

(13) $$D_{S^{c},U}^{F}=D_{S^{c},N}^{F}-\frac{\tau_{W}^{2}}{\sqrt{\sigma_{Y}^{2}+\tau_{W}^{2}}}\frac{\varphi \left\{\;f_{W}(X,Y)\right\}}{\Phi \left\{\;f_{W}(X,Y)\right\}},$$

where, after substituting *p*_*S*_ with *S*_*X*_/*n*_1_ in the expression for *f*_*W*_(*x*,*y*) given in Appendix B,

$$f_{W}(X,Y)=\left(\frac{\sqrt{\sigma_{Y}^{2}+\tau_{W}^{2}}}{\sigma_{Y}^{2}}\right)\left\{D_{S^{c},N}^{F}-\left(X-\frac{b}{1-S_{X}/n_{1}}\right)\right\}.$$

Consequently, an unbiased estimator for *θ*_*F*_ is $D_{\text{F, U}}=p_{S}D_{S,U}^{F}+p_{S^{c}}D_{S^{c},U}^{F}$, where $D_{S,U}^{F}$ and $D_{S^{c},U}^{F}$ are given by expressions (12) and (13), respectively.

### 2.4 Conditionally unbiased estimator for *θ* when the prevalence of the subpopulation is unknown

In the previous sections, we have assumed that *p*_*S*_, the true proportion of patients in *S*, is known. In some instances, this may not be a reasonable assumption. In this section, we derive conditionally unbiased estimator for *θ* when *p*_*S*_ is unknown. Unlike in Sections 2.2 and 2.3, for this case, *S*_*X*_ and *S*_*V*_ are random. We will assume *S*_*X*_, the number of patients from *S* in stage 1, is Binomial(*n*_1_,*p*_*S*_) so that consequently $\sigma_{X}^{2}$ and $\sigma_{Y}^{2}$ are now random. Define ${\hat{p}}_{S}=s_{X}/n_{1}$, where *s*_*X*_ is the observed value for *S*_*X*_ and $z^{*}={\hat{p}}_{S}x+\left(1-{\hat{p}}_{S}\right)y$. We assume that the trial continues to stage 2 with *S* if *x* > (*z* ^*^+*b*), which is equivalent to $x>y+b/\left(1-{\hat{p}}_{S}\right)$ and with *F* if $x\leq (z^{*}+b)$, which is equivalent to $x\leq y+b/\left(1-{\hat{p}}_{S}\right)$. Note that when *S* is selected, if we derive an estimator for *θ*_*S*_ that is unbiased conditional on *S*_*X*_=*s*_*X*_, then the estimator is unconditionally unbiased. We show in Appendix C that the UMVUE for *θ*_*S*_ when *S*_*X*_ is random is given by expression (11).

For the case where *F* is selected, we assume *S*_*V*_, the number of patients in *S* in stage 2, is Binomial(*n*_2_,*p*_*S*_) so that consequently $\tau_{V}^{2}$ and $\tau_{W}^{2}$ are now random. We show in Appendix D that the UMVUEs for *θ*_*S*_ and $\theta_{S^{c}}$ are given by expressions (12) and (13), respectively. Let

(14) $$D_{F,U}^{*}={\hat{p}}_{S}D_{S,U}^{F}+(1-{\hat{p}}_{S})D_{S^{c},U}^{F}.$$

As

$$E\;\left({\hat{p}}_{S}D_{S,U}^{F}\right)=\underset{s_{X}}{\sum }\left\{\frac{s_{X}}{n_{1}}\text{Prob}(S_{X}=s_{X})E(D_{S,U}|S_{X}=s_{X})\right\}=\theta_{S}\underset{s_{X}}{\sum }\frac{s_{X}}{n_{1}}\text{Prob}(S_{X}=s_{X})=p_{S}\theta_{S}$$

and similarly $E\;\left[\left(1-{\hat{p}}_{S}\right)D_{S^{c},U}^{F}\right]=\left(1-p_{S}\right)\theta_{S^{c}}$, $D_{F,U}^{*}$ is an unbiased estimator for *θ*_*F*_.

## 3 Worked example

In this section, we use an example to demonstrate how the various estimates described in Sections 2.2 and 2.3 are computed and how they compare. Computation of most estimates described in Section 2.4 would be similar to the computation of estimates in Section 2.3. Several trials for Alzheimer's disease (AD) consider continuous outcomes. In some AD trials, the primary outcome is continuous 30 so that our methodology can be used. Also, subgroup analysis has been considered in AD trials 20. Therefore, to construct the example, we use the AD trial reported in 31. This trial recruited patients with moderate or severe AD, with subgroup analysis performed later for patients with severe AD 20. We take the full population to consist of the patients with moderate or severe AD and the subpopulation to be the patients with severe AD that are thought to potentially benefit more from the new treatment. The primary outcome in 31 is not continuous and so for our example, we imagine that the primary outcome is Severe Impairment Battery (SIB) score, a 51‐item scale with scores ranging from 0 to 100. This was a secondary outcome in the original trial. For the AD trial in 31, the observed mean differences in SIB scores for patients with severe AD and the full population are 7.42 20 and 5.62 31, respectively. Based on these values, the observed mean difference for patients with moderate AD is approximately 3.82. Using the results for the severe AD patients, we will assume *σ* = 13.2. The AD trial 20 is single stage with approximately 290 patients. In the examples constructed here, we will assume a two‐stage ASD with *n*_1_=*n*_2_=200.

Using the definitions of Section 2.1, patients with severe AD form subpopulation *S*. Therefore, we denote the proportion and the true mean difference in SIB scores for patients with severe AD by *p*_*S*_ and *θ*_*S*_, respectively. In stage 1, the observed mean difference in SIB scores for patients with severe AD is denoted by *x*, and in stage 2, the observed mean difference in SIB scores for patients with severe AD is denoted by *u* if testing is only conducted for patients with severe AD and by *v* if the full population is tested. Also, from the definitions in Section 2.1, patients with moderate AD would form *S* ^*c*^ so that we denote the proportion and the true mean difference in SIB scores for patients with moderate AD by $p_{S^{c}}$ and $\theta_{S^{c}}$, respectively. In stage 1, the observed mean difference in SIB scores for patients with moderate AD is denoted by *y*, and in stage 2, if the full population is tested, we denote the observed mean difference in SIB scores for patients with moderate AD by *w*.

The proportion of patients with severe AD in 31 is approximately 0.5 so that for the example we take $p_{S}=p_{S^{c}}=0.5$. Because *n*_1_=*n*_2_=200 and *p*_*S*_=0.5 so that *S*_*X*_=*p*_*S*_ *n*_1_=100 and *S*_*V*_=*p*_*S*_ *n*_2_=100, using the definitions of Section 2.2, *t*_*S*_=*S*_*X*_/(*S*_*X*_+*n*_2_) = 1/3, $t_{S}^{F}=S_{X}/(S_{X}+S_{V})=0.5$ and $t_{S^{c}}^{F}=\left(n_{1}-S_{X}\right)/(n_{1}+n_{2}-S_{X}-S_{V})=0.5$. We assume the trial continues with *S* if the effect for patients with severe AD is greater than the effect for all patients so that *b* = 0 and *b*/(1 − *p*_*S*_) = 0. To compute the various unbiased estimates, we need $\sigma_{X}^{2}=4\sigma^{2}/S_{X}=6.97$ and $\sigma_{Y}^{2}=4\sigma^{2}/\left(n_{1}-S_{X}\right)=6.97$. If stage 2 data are only available for patients with severe AD, to compute an unbiased estimate for *θ*_*S*_, we need $\tau_{U}^{2}=4\sigma^{2}/n_{2}=3.48$. If the full population is tested in stage 2, to obtain the unbiased estimates for *θ*_*S*_ and $\theta_{S^{c}}$, we need $\tau_{V}^{2}=4\sigma^{2}/S_{V}=6.97$ and $\tau_{W}^{2}=4\sigma^{2}/(n_{2}-S_{V})=6.97$.

We will compute estimates for four scenarios. In the first two scenarios, *S* (patients with severe AD) is selected to continue to stage 2. In both scenarios, we suppose that *u* = 7.42. For Scenario 1, we suppose *x* = 6.5 and *y* = 5.6 and so the naive estimate for the mean difference for patients with severe AD, *d*_*S*,*N*_=*t*_*S*_ *x* + (1 − *t*_*S*_)*u* = 7.11. For the unbiased estimate, we use equation (11), with the unbiased estimate $d_{S,U}=d_{S,N}-\frac{\tau_{U}^{2}}{\sqrt{\sigma_{X}^{2}+\tau_{U}^{2}}}\frac{\varphi \{\;f_{U}(x,y)\}}{\Phi \{\;f_{U}(x,y)\}}$. The values for $\sigma_{X}^{2}$ and $\tau_{U}^{2}$ have been evaluated earlier and $f_{U}(x,y)=\left(\sqrt{\sigma_{X}^{2}+\tau_{U}^{2}}/\sigma_{X}^{2}\right)(d_{S,N}-y)=0.7$ so that *d*_*S*,*U*_=6.67. In the second scenario, we suppose *x* = 6.5 and *y* = 3.8 and using similar computation, *d*_*S*,*N*_=7.11 and *d*_*S*,*U*_=6.97. The naive estimates for Scenarios 1 and 2 are equal while the unbiased estimates are not equal, with the unbiased estimate for Scenario 2 closer to the naive estimate. Scenarios 1 and 2 differ in the values for *y* only and this is why the naive estimates are equal because, conditional on selecting *S*, the naive estimates depend on *x* and *u* only. However, the unbiased estimates depend on *y*, and as can be deduced from the expression for *f*_*V*_(*x*,*y*), acquire further from the naive estimate as the difference between the naive estimate and *y* decreases. This is reasonable because when data suggest that treatment effects for patients with moderate and severe AD are similar, selection bias is likely to be high. Same naive estimates and different unbiased estimates for Scenarios 1 and 2 may indicate more variability for the unbiased estimator for *θ*_*S*_ (*D*_*S*,*U*_) developed in Section 2.3 compared with the naive estimator *D*_*S*,*N*_.

The other two scenarios are for the case where the full population is tested in stage 2 and in both scenarios, we suppose that *v* = 7.42 and *w* = 3.48. For the third scenario, we suppose that *x* = 5.4 and *y* = 6.0 so that the naive estimates for *θ*_*S*_, $\theta_{S^{c}}$ and *θ*_*F*_ are $d_{S,N}^{F}=t_{S}^{F}x+\left(1-t_{S}^{F}\right)v=6.41$, $d_{S^{c},N}^{F}=t_{S^{c}}^{F}y+\left(1-t_{S^{c}}^{F}\right)w=4.91$ and $d_{F,N}=p_{S}d_{S,N}+p_{S^{c}}d_{S^{c},N}^{F}=5.66$, respectively. Using equations (12) and (13), the unbiased estimates for *θ*_*S*_ and $\theta_{S^{C}}$ are given by $d_{S,U}^{F}=d_{S,N}^{F}+\frac{\tau_{V}^{2}}{\sqrt{\sigma_{X}^{2}+\tau_{V}^{2}}}\frac{\varphi \left\{\;f_{V}(x,y)\right\}}{\Phi \left\{\;f_{V}(x,y)\right\}}$ and $d_{S^{c},U}^{F}=d_{S^{c},N}^{F}-\frac{\tau_{W}^{2}}{\sqrt{\sigma_{Y}^{2}+\tau_{W}^{2}}}\frac{\varphi \left\{\;f_{W}(x,y)\right\}}{\Phi \left\{\;f_{W}(x,y)\right\}}$, respectively, where $f_{V}(x,y)=\left(\sqrt{\sigma_{X}^{2}+\tau_{V}^{2}}/\sigma_{X}^{2}\right)\left(\;y-d_{S,N}^{F}\right)=-0.22$ and $f_{W}(x,y)=\left(\sqrt{\sigma_{Y}^{2}+\tau_{W}^{2}}/\sigma_{Y}^{2}\right)(d_{S^{c},N}^{F}-x)=-0.26$. Consequently, $d_{S,U}^{F}=8.17$, $d_{S^{c},U}^{F}=3.10$ and the unbiased estimate for *θ*_*F*_, $d_{F,U}=p_{S}d_{S,U}^{F}+p_{S^{c},U}d_{S^{c},U}^{F}=5.63$. The corresponding naive and unbiased estimates are not equal. In the fourth scenario, we suppose *x* = 5.7 and *y* = 5.7 and using similar formulae as in Scenario 3, $d_{S,N}^{F}=6.56$, $d_{S^{c},N}^{F}=4.76$, *d*_*F*,*N*_=5.66, $d_{S,U}^{F}=8.64$, $d_{S^{c},U}^{F}=2.62$, *d*_*F*,*U*_=5.63. In both scenarios, the naive estimates for *θ*_*F*_ are equal. The unbiased estimates for *θ*_*F*_ in Scenarios 3 and 4 are also equal. However, the corresponding naive estimates for *θ*_*S*_ and $\theta_{S^{c}}$ in Scenarios 3 and 4 are different. The corresponding unbiased estimates for *θ*_*S*_ and $\theta_{S^{c}}$ in Scenarios 3 and 4 are also different. For both *θ*_*S*_ and $\theta_{S^{c}}$, the difference between the naive estimates in Scenarios 3 and 4 is greater than the difference between the unbiased estimates in Scenarios 3 and 4. This is because the unbiased estimators for *θ*_*S*_ and $\theta_{S^{c}}$ are functions of all stage 1 data while the naive estimators for *θ*_*S*_ and $\theta_{S^{c}}$ only use data from populations *S* and *S* ^*c*^, respectively. The larger differences between the unbiased estimates in Scenarios 3 and 4 than the differences between the naive estimates, may indicate more variability for the unbiased estimator for *θ*_*F*_ (*D*_*F*,*U*_) developed in Section 2.3 than the naive estimator *D*_*F*,*N*_. In Scenario 4, compared with Scenario 3, $d_{S,U}^{F}$ and $d_{S^{c},U}^{F}$ are further from $d_{S,N}^{F}$ and $d_{S^{c},N}^{F}$, respectively. This is reasonable because although in both scenarios $d_{S^{c},N}^{F}$ is smaller than $d_{S,N}^{F}$ so that the observed data suggest a correct decision for both scenarios would be to continue to stage 2 with patients with severe AD only, in Scenario 4, $d_{S^{c},N}^{F}$ is much smaller than $d_{S,N}^{F}$ providing more evidence that the correct decision would have been to continue with patients with severe AD only, and hence more adjustments to the naive estimates are required.

## 4 Comparison of the estimators

In this section, we assess the bias of the naive estimator and use a simulation study to compare the mean squared errors for the naive and unbiased estimators for several scenarios.

### 4.1 Characteristics of the calculated bias for the naive estimator

**Figure 2:**
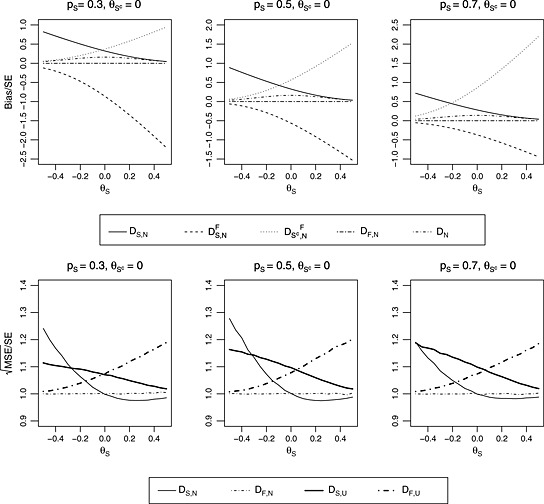
Plots showing bias (top row) and mean squared error (bottom row) for the case where *n*_1_=*n*_2_=200, *σ* ^2^=1 and $\theta_{S^{c}}=0$. The *x*‐axes correspond to the values for *θ*_*S*_. Each column corresponds to a different value for *p*_*S*_. MSE, mean squared error; SE, standard error.

From the bias functions derived in Section 2.2, we note that the bias for the naive estimator depends on *θ*_*S*_, $\theta_{S^{c}}$ and *p*_*S*_ and so we will vary the values of these parameters. The bias also depends on *t*_*F*_=*n*_1_/(*n*_1_+*n*_2_) but we will only present results for the scenario where *n*_1_=*n*_2_=200 so that *t*_*F*_=0.5. From the expressions for biases, one can demonstrate that biases increase as one makes selection later in the trial, that is, as *t*_*F*_ increases. The other parameters that bias depends on are *b*, *σ* ^2^ and *n*_1_+*n*_2_. In this section, we will take *σ* ^2^=1. For a given value of *t*_*F*_, to make the results approximately invariant of *σ* ^2^ and *n*_1_+*n*_2_, we will divide *D*_*S*,*N*_ by $\sqrt{4/\left(\;p_{S}n_{1}+n_{2}\right)}$, which is the approximate standard error (SE) for *D*_*S*,*N*_ and we will divide estimators $D_{S^{c},N}^{F}$, $D_{S,N}^{F}$ and *D*_*F*,*N*_ by $\sqrt{4/(n_{1}+n_{2})}$, which is the approximate SE for *D*_*F*,*N*_. We will comment how *b* influences bias after describing results in Figure 2 for which *b* = 0. The top row in Figure 2 explores the bias for the naive estimator and how the naive estimators for treatment effects in *S* and *S* ^*c*^ contribute to the bias. From left to right, the plots correspond to *p*_*S*_=0.3, *p*_*S*_=0.5 and *p*_*S*_=0.7. The *y*‐axes give the biases. The *x*‐axes correspond to different values for *θ*_*S*_ and in all plots, $\theta_{S^{c}}=0$. We have taken a fixed value for $\theta_{S^{c}}$ because bias depends on *θ*_*S*_ and $\theta_{S^{c}}$ only through $\left(\theta_{S}-\theta_{S^{c}}\right)$. This can be observed by noting that if we add some value *δ* to *θ*_*S*_ and $\theta_{S^{c}}$, the expressions for bias in Section 2.2 change by having *t* − *δ* − *θ*_*S*_, $t-\delta -\theta_{S^{c}}$, $t-\delta -\theta_{S}^{*}$ and $t-\delta -\theta_{S^{c}}^{*}$ in place of *t* − *θ*_*S*_, $t-\theta_{S^{c}}$, $t-\theta_{S}^{*}$ and $t-\theta_{S^{c}}^{*}$, respectively. If we let *r* = *t* − *δ* and integrate with respect to *r* and use subscript *δ* for the new expressions used to obtain bias, these can be expressed as Pr_*δ*_(*X* > *Y* ^*^) = Pr(*X* > *Y* ^*^), ${\mathrm{Pr}}_{\delta }\left(X\leq Y^{*}\right)=\mathrm{Pr}\left(X\leq Y^{*}\right)$, $E_{\delta }\left(X1_{\left[X>Y^{*}\right]}\right)=E\;\left(X1_{\left[X>Y^{*}\right]}\right)+\delta \mathrm{Pr}\left(X>Y^{*}\right),E_{\delta }\left(\;Y1_{\left[X\leq Y^{*}\right]}\right)=E\;\left(\;Y1_{\left[X\leq Y^{*}\right]}\right)+\delta \mathrm{Pr}\left(X\leq Y^{*}\right)$ and $E_{\delta }\left(X1_{\left[X\leq Y^{*}\right]}\right)=E\left(X1_{\left[X\leq Y^{*}\right]}\right)+\delta \mathrm{Pr}\left(X\leq Y^{*}\right)$. Substituting the new expressions in equations (5) and (10), we obtain the same forms for bias and hence the same bias when *δ* is added to both *θ*_*S*_ and $\theta_{S^{c}}$.

In Figure 2, the legends at the bottom of the plots describe the line types for each estimator. In the first legend, the continuous lines (—) correspond to the case where *S* is selected to continue to stage 2 and hence gives the bias for *D*_*S*,*N*_ as an estimator for *θ*_*S*_. The bias for *D*_*S*,*N*_ decreases as $\left(\theta_{S}-\theta_{S^{c}}\right)$ increases. This is reasonable because as *θ*_*S*_ becomes larger than $\theta_{S^{c}}$, Pr(*X* > *Y* ^*^) approaches 1 so that the density of *X* conditional on *X* > *Y* ^*^ approaches the unconditional density of *X* and consequently the bias for *D*_*S*,*N*_ approaches zero. The decrease of bias for *D*_*S*,*N*_ can also be explained by the expressions for Pr(*X* > *Y* ^*^) and $E\;\left(X1_{\left[X>Y^{*}\right]}\right)$ that are given by equations (6) and (7), respectively. As *θ*_*S*_ becomes larger than $\theta_{S^{c}}$, the density for the sample mean difference in *S* becomes stochastically larger than the density for the sample mean difference in *S* ^*c*^ and consequently, for the values of *t*, where the term with *φ* in Pr(*X* > *Y* ^*^) and $E\;\left(X1_{\left[X>Y^{*}\right]}\right)$ is non‐zero, the term with Φ approaches 1. Hence, Pr(*X* > *Y* ^*^) and $E\;\left(X1_{\left[X>Y^{*}\right]}\right)$ approach one and *E*(*X*), respectively so that the bias approaches zero. The dotted lines (···) show the bias for $D_{S^{c},N}^{F}$, the naive estimator for $\theta_{S^{c}}$ when *F* is selected. The bias is positive and increases as $\left(\theta_{S}-\theta_{S^{c}}\right)$ increases. The dashed lines (‐ ‐ ‐) show the bias for $D_{S,N}^{F}$, the naive estimator for *θ*_*S*_ when *F* is selected. The bias is negative and increases as $\left(\theta_{S}-\theta_{S^{c}}\right)$ increases. The explanation for the behaviours of the biases for $D_{S^{c},N}^{F}$ and $D_{S,N}^{F}$ is similar to the explanation for the behaviour of the bias for *D*_*S*,*N*_. When *p*_*S*_=0.5 (middle panel), except for the sign, the bias for $D_{S,N}^{F}$ is equal to the bias for $D_{S^{c},N}^{F}$. For the other values for *p*_*S*_ (other panels in Figure 2), except for the sign, we note that $\text{Bias}(D_{S,N}^{F})$ multiplied by *p*_*S*_ is equal to $\text{Bias}(D_{S^{c},N}^{F})$ multiplied by $p_{S^{c}}$. Therefore, if *F* is selected, although the naive components $D_{S,N}^{F}$ and $D_{S^{c},N}^{F}$ are biased, as can be seen from the short and long dashed lines (– ‐ – ‐ –), the naive estimator *D*_*F*,*N*_ is unbiased. The dashed and dotted line (·−·−·) shows the bias for *D*_*N*_, the naive estimator for *θ*. The bias is maximal when $\theta_{S}=\theta_{S^{c}}$. Based on results not presented here, for *b* ≠ 0, the lines in Figure 2 shift by *b*/(1 − *p*_*S*_) so that bias is maximal when $\theta_{S}=\theta_{S^{c}}+b/\left(1-p_{S}\right)$. Noting that the selection is based on max{*X*,*Y* + *b*/(1 − *p*_*S*_)}, the proof that the bias is maximal when $\theta_{S}=\theta_{S^{c}}+b/\left(1-p_{S}\right)$ is given by Carreras and Brannath 32.

From the aforementioned assessment of the bias for the naive estimator, we note that, when *S* is selected, the bias for the naive estimator for *θ*_*S*_ is substantial. If *F* is selected, the naive estimator for *θ*_*F*_ is unbiased. However, the naive estimators for *θ*_*S*_ and $\theta_{S^{c}}$ are substantially biased. It is our view that we need unbiased estimators for *θ*_*S*_ and $\theta_{S^{c}}$ such as those developed in Section 2.3 when *F* is selected because we believe investigators would still want to learn about *θ*_*S*_ and $\theta_{S^{c}}$.

### 4.2 Simulation of mean squared errors

In this section, we perform a simulation study to compare the MSEs for the naive and unbiased estimators. In Section 4.1, for the case where *F* is selected, as well as exploring the bias for the naive estimator for *θ*_*F*_, we have also explored the bias for the naive estimators for *θ*_*S*_ and $\theta_{S^{c}}$, which are the components for *θ*_*F*_. In this section, we will only focus on the estimators that will be used for inference after stage 2. Hence, when *F* is selected, we will only compare MSEs for the naive and unbiased estimators for *θ*_*F*_, and when *S* is selected, we will only compare MSEs for the naive and unbiased estimators for *θ*_*S*_. For each combination of *θ*_*S*_, $\theta_{S^{c}}$, *t*_*F*_ and *p*_*S*_, we run 1,000,000 simulation runs. As in Section 4.1, we will only present the MSE results for the case where *b* = 0. For simulations with *b* = 0, in each simulation run, we simulate stage 1 data (*x* and *y*) and if *x* > *y* in which case *S* would be selected to continue to stage 2, we simulate *u* and if x≤y in which case *F* would be selected to continue to stage 2, we simulate *v* and *w*.

The bottom row in Figure 2 gives the square root of the MSEs divided by approximate SE. As indicated earlier, these plots are for the cases where *b* = 0. Based on results not presented here, for *b* ≠ 0, the lines in Figure 2 shift by *b*/(1 − *p*_*S*_). For both the naive and unbiased estimators, we take $\text{SE}=\sqrt{4/\left(\;p_{S}n_{1}+n_{2}\right)}$ when *S* is selected to continue to stage 2 and $\text{SE}=\sqrt{4/\left(n_{1}+n_{2}\right)}$ when *F* is selected to continue to stage 2. From left to right, *p*_*S*_=0.3, *p*_*S*_=0.5 and *p*_*S*_=0.7, respectively. The second legend at the bottom of the plots describes the line types for each estimator. The continuous lines (—) correspond to *D*_*S*,*N*_, the naive estimator for *θ*_*S*_ when *S* is selected to continue to stage 2. The MSE for *D*_*S*,*N*_ decreases as $\left(\theta_{S}-\theta_{S^{c}}\right)$ increases and varies with the values for *p*_*S*_ but not monotonically. The thick continuous lines (**—**) correspond to *D*_*S*,*U*_, the UMVUE for *θ*_*S*_ when *S* is selected to continue to stage 2. The MSE for *D*_*S*,*U*_ decreases as $\left(\theta_{S}-\theta_{S^{c}}\right)$ increases and seems to increase as the values for *p*_*S*_ increase. For most scenarios, the MSE for *D*_*S*,*U*_ is larger than the MSE for *D*_*S*,*N*_. As for bias, the dashed and dotted lines (·−·−·) correspond to *D*_*F*,*N*_, the naive estimator for *θ*_*F*_ when *F* is selected to continue to stage 2 and for all scenarios, the $\sqrt{\text{MSE}(D_{F,N})}/\mathrm{SE}$ is approximately 1. The thick dashed and dotted lines (**‐** **·** **‐**·**‐**) correspond to *D*_*F*,*U*_, the unbiased estimator for *θ*_*F*_ when *F* is selected to continue to stage 2. The MSE for *D*_*F*,*U*_ increases with $\left(\theta_{S}-\theta_{S^{c}}\right)$ and *p*_*S*_.

For the case where *S* is selected to continue to stage 2, comparing the biases and MSEs for *D*_*S*,*N*_ and *D*_*S*,*U*_, we would recommend using *D*_*S*,*U*_. This is because although for most scenarios in Figure 2, the MSE for *D*_*S*,*U*_ is greater than the MSE for *D*_*S*,*N*_, the gain achieved by *D*_*S*,*U*_ being an unbiased estimator outweighs the loss of precision by using *D*_*S*,*U*_. For example, from the results in the top left and bottom left plots, when *θ*_*S*_=0, (Bias(*D*_*S*,*N*_))/*S* *E* is 0.32 while $\sqrt{\text{MSE}(D_{S,N})}/\mathrm{SE}$ is 0.07 less than $\sqrt{\text{MSE}(D_{S,U})}/\mathrm{SE}$ so that *D*_*S*,*U*_ removes substantial bias at the expense of a slight loss of precision around the true treatment effect. Similar results are observed in the other plots. For the case where *F* is selected to continue to stage 2, from the results in Figure 2, *D*_*F*,*N*_ seems a better estimator for *θ*_*F*_ than the estimator *D*_*F*,*U*_ because both are mean unbiased but *D*_*F*,*N*_ has smaller MSE.

The summary findings from the simulation study is that bias for the naive estimators can be substantial but the naive estimators have lower MSEs than the unbiased estimators we derived in Section 2.3. Balancing between the gain of having an unbiased estimator and the loss of precision, when *S* is selected, we recommend using the unbiased estimator for *θ*_*S*_ given by expression (11). When *F* is selected, both the naive estimator *D*_*F*,*N*_ and the unbiased estimator *D*_*F*,*U*_ are mean unbiased but *D*_*F*,*N*_ has better precision than *D*_*F*,*U*_ and so for the case when *F* is selected, we recommend using the naive estimator for *θ*_*F*_ (*D*_*F*,*N*_) given by expression (8).

### 4.3 Properties of the estimators when the prevalence of the subpopulation is unknown

The results in Sections 4.1 and 4.2 are for the case of known *p*_*S*_. In this section, we assess the performance of the various estimators when *p*_*S*_ is unknown. To do this, we use the true value *p*_*S*_ to simulate the number of patients in *S* in stage 1 (*s*_*X*_) as Binomial(*n*_1_,*p*_*S*_) and calculate ${\hat{p}}_{S}=s_{X}/n_{1}$. After simulating *s*_*X*_, we then simulate stage 1 sample mean differences *x* and *y* for populations *S* and *S* ^*c*^, respectively. As in Sections 4.1 and 4.2, we only present results for the case where *b* = 0. For this case, because $b/\left(1-{\hat{p}}_{S}\right)=0$, we select *S* if *x* > *y* and select *F* if x≤y. If *S* is selected, we simulate stage 2 sample mean difference *u* from a sample consisting of *n*_2_/2 patients in each of the control and experimental arms. The naive estimate for *θ*_*S*_ is *d*_*S*,*N*_=(*s*_*X*_ *x* + *n*_2_ *u*)/(*s*_*X*_+*n*_2_). The unbiased estimate for *θ*_*S*_, *d*_*S*,*U*_, is obtained using expression (11). If *F* is selected, we use the true value *p*_*S*_ to simulate the number of patients in *S* in stage 2 (*s*_*V*_) as Binomial(*n*_2_,*p*_*S*_). For each of *S* and *S* ^*c*^, we assume the number of patients are equally allocated to the control and the experimental treatments. Based on *s*_*V*_ patients from *S* and (*n*_2_−*s*_*V*_) patients from *S* ^*c*^, we simulate stage 2 sample mean differences *v* and *w* for *S* and *S* ^*c*^, respectively. Let ${\hat{p}}_{S}^{*}=(s_{X}+s_{V})/(n_{1}+n_{2})$, we compute the naive estimates for *θ*_*S*_, $\theta_{S^{c}}$ and *θ*_*F*_ as $d_{S,N}^{F}=t_{S}^{F}x+\left(1-t_{S}^{F}\right)v$, $d_{S^{c},N}^{F}=t_{S^{c}}^{F}y+\left(1-t_{S^{c}}^{F}\right)w$ and $d_{F,N}^{*}={\hat{p}}_{S}^{*}d_{S,N}^{F}+\left(1-{\hat{p}}_{S}^{*}\right)d_{S^{c},N}^{F}$, respectively. Note that when *p*_*S*_ is known ${\hat{p}}_{S}^{*}=p_{S}$ so that the estimator $D_{F,N}^{*}$ can be reasonably compared with the estimator *D*_*F*,*N*_ given by expression (8). The unbiased estimates for $\theta_{S}\left(d_{S,U}^{F}\right)$ and $\theta_{S^{c}}\left(d_{S^{c},U}^{F}\right)$ are obtained using expressions (12) and (13), respectively. The unbiased estimate for *θ*_*F*_ is calculated as $d_{F,U}={\hat{p}}_{S}d_{S,U}^{F}+\left(1-{\hat{p}}_{S}\right)d_{S^{c},U}^{F}$.

**Figure 3:**
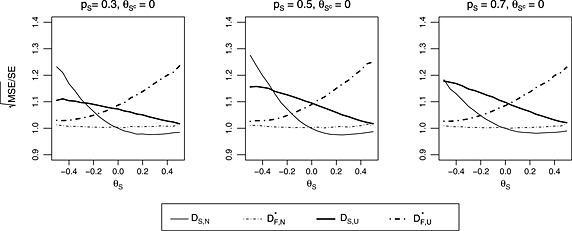
Mean squared error for the case where *n*_1_=*n*_2_=200, *σ* ^2^=1 and $\theta_{S^{c}}=0$. The x‐axes correspond to the values for *θ*_*S*_. Each column corresponds to a different value for *p*_*S*_. MSE, mean squared error; SE, standard error.

Figure 3 gives the simulation results for the configurations considered in Figure 2. The form of the SEs used in Figure 3 are the same as those used in Figure 2. We have not presented bias plots because the estimators obtained assuming that *p*_*S*_ is known, have very similar biases to the estimators obtained assuming that *p*_*S*_ is unknown. When *S* is selected, for the naive estimator for *θ*_*S*_, there is no noticeable difference in MSEs between the case when *p*_*S*_ is assumed known and the case when *p*_*S*_ is assumed unknown so that *S*_*X*_ is random. Similar results are observed for *D*_*S*,*U*_, the unbiased estimator for *θ*_*S*_ when *S* is selected. For the case where *F* is selected, for both the naive estimator and the unbiased estimator for *θ*_*F*_, the MSEs for the case where *p*_*S*_ is assumed known and the case where *p*_*s*_ is estimated are approximately equal when $\theta_{S}=\theta_{S^{c}}$. When $\theta_{S}\neq \theta_{S^{c}}$, the MSEs for $D_{F,N}^{*}$ and $D_{F,U}^{*}$ for the case where *p*_*S*_ is estimated are slightly higher than MSEs for *D*_*F*,*N*_ and *D*_*F*,*U*_, respectively, which are the estimators for the case where *p*_*S*_ is assumed known. Based on results not presented here, when *b* ≠ 0 so that we select *S* if $x>y+b/\left(1-{\hat{p}}_{S}\right)$ and select *F* if $x\leq y+b/\left(1-{\hat{p}}_{S}\right)$, as in the case where *p*_*s*_ is assumed known, we noted that the lines in Figure 3 shift by *b*/(1 − *p*_*S*_).

To summarise, the results obtained when *p*_*S*_ is estimated are very similar to results when *p*_*S*_ is assumed known. The biases for the different estimators for *θ*_*S*_ and *θ*_*F*_ are almost identical and MSEs are only slightly higher. The reason that the increases in MSEs are not substantial for the case when *p*_*S*_ is estimated may be as a result of adequate sample size in stage 1 and hence good precision for the estimator for *p*_*S*_. An estimator for *p*_*S*_ with good precision would not add a great deal of variability to the estimators for *θ*_*S*_ and *θ*_*F*_. Thus, if stage 1 data are adequate to estimate *p*_*S*_, the estimators developed in this paper perform almost as good as when *p*_*S*_ is known.

## 5 Discussion

In order to make testing of new interventions more efficient, ASDs have been proposed. Such designs have been used for trials with subpopulation selection. This is the case we consider in this paper. Specifically, we have considered a design that has two stages, with data collected from stage 1 used to select the population to test in stage 2. In stage 2, additional data for a sample drawn from the selected population are collected. The final confirmatory analysis uses data from both stages. Statistical methods that have previously been developed to adjust for selection bias that arise from using stage 1 data have addressed hypothesis testing without inflating type I error. In this work, we have focussed on point estimation. We have derived formulae for obtaining unbiased point estimators. We have derived the formulae for the case where the prevalence of the subpopulation is considered known and also for the case where the prevalence of the subpopulation is unknown. To acquire unbiased estimators when the prevalence of the subpopulation is unknown, we have derived formulae for unbiased estimators when the proportion of patients from the subpopulation does not have to be equal to the prevalence of the subpopulation. This means that the estimators we have derived can be used to obtain unbiased estimates for trials that use enrichment designs, where proportion of the subpopulation in the trial is not equal to the prevalence of the subpopulation. The rest of this discussion focusses on the case where the prevalence of the subpopulation is assumed known but most points also hold for the case where the prevalence is unknown.

The unbiased estimators we have developed have higher MSEs compared with the naive estimators. Balancing between unbiasedness and precision, when the subpopulation is selected, we recommend using the unbiased estimator we have derived and when the full population is selected, we recommend using the naive estimator. The unbiased estimator for *θ*_*F*_ that we derived conditional on continuing to stage 2 with the full population, although based on UMVUEs for *θ*_*S*_ and $\theta_{S^{c}}$, may not be a UMVUE among estimators for *θ*_*F*_ that are functions of unbiased estimators for *θ*_*S*_ and $\theta_{S^{c}}$ and so more research is required to check whether it is an UMVUE and if not, seek an UMVUE.

The estimators we have developed in this paper are unbiased conditional on the selection made. For the case where the full population is selected, we have derived separate unbiased estimators for the treatment effects for the subpopulation and its complement. These estimators are unbiased only if we do not make a selection after stage 2. That is, for the case where the full population continues to stage 2, if we use the observed separate estimates to make a claim that the treatment effect is larger in the subpopulation or in the full population, then the estimators developed in this paper are no longer unbiased. In this case, the same data are used both for selection and estimation, and Stallard *et al*. 33 have shown that there is no unbiased estimator.

We have considered the case whether the selection rule is pre‐defined and based on the efficacy outcome. In terms of estimation, a pre‐defined selection rule makes it possible to derive point estimators and evaluate their biases because bias is an expectation, and it is not clear what all possible outcomes are when the selection rule is not pre‐defined. The Food and Drug Administration draft guidance also acknowledges the difficulty in interpreting trial results when adaptation is not pre‐defined 34. A compromise between a pre‐defined selection rule used in this paper and a setting where the selection rule is not pre‐defined is a pre‐defined selection rule that includes additional aspects such as safety. More work is required to develop point estimators for such settings.

The unbiased estimators we have developed are for the case where the subpopulations are pre‐specified and they cannot be assumed to be unbiased in trials where subpopulations are not pre‐specified. It is flexible not to pre‐specify subpopulations but it is hard to evaluate bias of point estimators because bias is an expectation, and it is not clear what all possible outcomes are when the subpopulations are not pre‐specified. Hence, it is not possible to quantify the bias of the estimators developed here when the subpopulations are not pre‐specified 35.

Depending on the number of subpopulations and how they are defined, there are several configurations on how the subpopulations can be nested within each other 25. We have focussed on a simple and common configuration where a single subpopulation is thought to benefit more, so that based on stage 1 data, the investigators want to choose between continuing with the full population or the subpopulation. By noting that to obtain unbiased estimators we have partitioned the full population into distinct parts, the formulae we have developed for this configuration can be extended to other configurations. If the full population is not of interest and the other subpopulations are not nested with each other, the subpopulations already form distinct parts and the formulae derived for treatment selection such as in 15 can be used directly. If the full population is of interest or some subpopulations are nested within each other, it is possible to partition the full population into distinct parts and use our methodology to obtain unbiased estimators for the distinct parts. However, following our findings that for some selections the naive estimator is unbiased and has better precision than an estimator that combines unbiased estimators for the distinct parts, in order to make a recommendation on the best estimator for the case where there is nesting of subpopulations, we suggest comparing the characteristics of the naive estimators to the estimator that combines unbiased estimators for the distinct parts in the population.

We have assumed that whether the subpopulation or the full population is selected, the total sample size in stage 2 is fixed. The results also hold for the case where stage 2 sample sizes for continuing with the subpopulation and the full population are different but prefixed for each selection made. The results may not hold when the stage 2 sample size depends on the observed data in some other way.

Finally, if there is a futility rule that requires the trial to continue to stage 2 only if the mean difference for the selected population exceeds some pre‐specified value and as in 15 estimation is conditional on continuing to stage 2, the unbiased estimators developed in Section 2.3 can be extended to account for this. If we denote the pre‐specified futility value by *B* so that the trial stops if max{*x*,*z*}<*B*, the expression for *f*_*U*_(*X*,*Y*) in equation (11) becomes $\left(\sqrt{\sigma_{X}^{2}+\tau_{U}^{2}}/\sigma_{X}^{2}\right)\left[D_{S,N}-\max\left\{B,\left(Y+b/\left(1-p_{S}\right)\right)\right\}\right]$ and the expression for *f*_*W*_(*X*,*Y*) in equation (13) becomes $\left(\sqrt{\sigma_{Y}^{2}+\tau_{W}^{2}}/\sigma_{Y}^{2}\right)\left[D_{S^{c},N}^{F}-\max\left\{\left(X-b/\left(1-p_{S}\right)\right),(B-p_{S}X)/\left(1-p_{S}\right)\right\}\right]$. The expression given by equation (12) changes to

$$D_{S,U}^{F}=D_{S,N}^{F}-\frac{\tau_{V}^{2}}{\sqrt{\sigma_{X}^{2}+\tau_{V}^{2}}}\frac{\varphi \left\{\;f_{V}(X,Y)\right\}-\varphi \left\{\;f_{V_{B}}(X,Y)\right\}}{\Phi \left\{\;f_{V}(X,Y)\right\}-\Phi \left\{\;f_{V_{B}}(X,Y)\right\}},$$

where *f*_*V*_(*X*,*Y*) is as given before, and the expression for $f_{V_{B}}(X,Y)$ is $\left(\sqrt{\sigma_{X}^{2}+\tau_{V}^{2}}/\sigma_{X}^{2}\right)$ $\left[(B-\left(1-p_{S}\right)Y)/p_{S}-D_{S,N}^{F}\right]$. Note that with a futility rule, the naive estimator for *θ*_*F*_ defined in this paper is no longer mean unbiased.

## Appendix A Uniformly minimum variance unbiased estimator for *θ*_*S*_ when the subpopulation is selected and *p*_*S*_ is known

### A.1

Here, we derive the UMVUE for *θ*_*S*_ conditional on selecting *S*. We will skip several steps that are similar to the steps used in 11, 14, 15. The notation used in 15 is very similar to the notation we have used. Let *Q*_*S*_ denote the event *X* > *Y* + *b*/(1 − *p*_*S*_). Conditional on the event *Q*_*S*_, the density *f*(*u*,*x*,*y*) is given by

$$\frac{1}{\tau_{U}}\varphi \left(\frac{u-\theta_{S}}{\tau_{U}}\right)\frac{1}{\sigma_{X}}\varphi \left(\frac{x-\theta_{S}}{\sigma_{X}}\right)\frac{1}{\sigma_{Y}}\varphi \left(\frac{y-\theta_{S^{c}}}{\sigma_{Y}}\right)1_{Q_{S}}K_{S}^{-1}(\theta ),$$

where $1_{Q_{S}}$ denotes the indicator function for the event *Q*_*S*_ and *K*_*S*_(*θ*) the probability for event *Q*_*S*_ given the true parameter vector $\theta =\left(\theta_{S},\theta_{S^{c}}\right)$. The density *f*(*u*,*x*,*y*) can be re‐expressed to

$$\frac{1}{\tau_{U}}\varphi \left(\frac{\frac{\tau_{U}}{\sigma_{X}}x+\frac{\sigma_{X}}{\tau_{U}}u-\theta_{S}\alpha_{1}}{\sqrt{\sigma_{X}^{2}+\tau_{U}^{2}}}\right)\frac{1}{\sigma_{X}}\varphi \left(\frac{x-\left(\frac{\tau_{U}}{\sigma_{X}}x+\frac{\sigma_{X}}{\tau_{U}}u\right)/\alpha_{1}}{\frac{\sigma_{X}^{2}}{\tau_{U}^{2}}\alpha_{2}}\right)\frac{1}{\sigma_{Y}}\varphi \left(\frac{y-\theta_{S^{c}}}{\sigma_{Y}}\right)1_{Q_{S}}K_{S}^{-1}(\theta ),$$

where

$$\alpha_{1}=\frac{\sigma_{X}}{\tau_{U}}+\frac{\tau_{U}}{\sigma_{X}}\;\;\;\text{and}\;\;\;\alpha_{2}=\frac{\tau_{U}^{2}}{\sqrt{\sigma_{X}^{2}+\tau_{U}^{2}}}.$$

Let *z*_*s*_=(*τ*_*U*_/*σ*_*X*_)*x* + (*σ*_*X*_/*τ*_*U*_)*u*; from the preceding density, conditional on *Q*_*S*_, (*Y*,*Z*_*S*_) is sufficient and complete statistic for estimating *θ*_*S*_. Therefore, because conditional on *Q*_*S*_, *U* is an unbiased estimator for *θ*_*S*_, the UMVUE for *θ*_*S*_ is *E*[*U*|*Y*,*Z*_*S*_,*Q*_*S*_]. To obtain the expression for *E*[*U*|*Y*,*Z*_*S*_,*Q*_*S*_], we derive the density *f*(*u*|*y*,*z*_*S*_,*Q*_*S*_) = *f*(*u*,*y*,*z*_*S*_|*Q*_*S*_)/*f*(*y*,*z*_*S*_|*Q*_*S*_).

The aforementioned density can be transformed to obtain the density *f*(*x*,*y*,*z*_*S*_|*Q*_*S*_) given by

$$\varphi \left(\frac{z_{S}-\theta_{S}\alpha_{1}}{\sqrt{\sigma_{X}^{2}+\tau_{U}^{2}}}\right)\frac{1}{\sigma_{X}^{2}}\varphi \left(\frac{x-z_{S}/\alpha_{1}}{\frac{\sigma_{X}^{2}}{\tau_{U}^{2}}\alpha_{2}}\right)\frac{1}{\sigma_{Y}}\varphi \left(\frac{y-\theta_{S^{c}}}{\sigma_{Y}}\right)1_{Q_{S}}K_{S}^{-1}(\theta ),$$

and to obtain the density *f*(*u*,*y*,*z*_*S*_|*Q*_*S*_) given by

$$\varphi \left(\frac{z_{S}-\theta_{S}\alpha_{1}}{\sqrt{\sigma_{X}^{2}+\tau_{U}^{2}}}\right)\frac{1}{\tau_{U}^{2}}\varphi \left(\frac{u-z_{S}/\alpha_{1}}{\alpha_{2}}\right)\frac{1}{\sigma_{Y}}\varphi \left(\frac{y-\theta_{S^{c}}}{\sigma_{Y}}\right)1_{Q_{S}}K_{S}^{-1}(\theta ).$$

The density *f*(*y*,*z*_*S*_|*Q*_*S*_) is obtained from *f*(*x*,*y*,*z*_*S*_|*Q*_*S*_) by integrating out *x* as follows

(A.1) $$\begin{array}{cc} f(\;y,z_{S}|Q_{S}) & =\frac{1}{\sigma_{X}^{2}}\varphi \left(\frac{z_{S}-\theta_{S}\alpha_{1}}{\sqrt{\sigma_{X}^{2}+\tau_{U}^{2}}}\right)\frac{1}{\sigma_{Y}}\varphi \left(\frac{y-\theta_{S^{c}}}{\sigma_{Y}}\right)\left\{\overset{\infty }{\underset{y+b/\left(1-p_{S}\right)}{\int }}\varphi \left(\frac{x-z_{S}/\alpha_{1}}{\frac{\sigma_{X}^{2}}{\tau_{U}^{2}}\alpha_{2}}\right)\mathrm{dx}\right\}1_{Q_{S}}K_{S}^{-1}(\theta )\\ =\frac{\alpha_{2}}{\tau_{U}^{2}}\varphi \left(\frac{z_{S}-\theta_{S}\alpha_{1}}{\sqrt{\sigma_{X}^{2}+\tau_{U}^{2}}}\right)\frac{1}{\sigma_{Y}}\varphi \left(\frac{y-\theta_{S^{c}}}{\sigma_{Y}}\right)[1-\Phi \{-f_{U}(x,y)\}]1_{Q_{S}}K_{S}^{-1}(\theta ), \end{array}$$

where

$$f_{U}(x,y)\;=\;\left(\sqrt{\sigma_{X}^{2}+\tau_{U}^{2}}/\sigma_{X}^{2}\;\right)\;\;\left\{\;\;\frac{\tau_{U}^{2}x+\sigma_{X}^{2}u}{\sigma_{X}^{2}+\tau_{U}^{2}}-\left(\;y+\frac{b}{1-p_{S}}\right)\;\;\right\}\;=\;\left(\;\sqrt{\sigma_{X}^{2}+\tau_{U}^{2}}/\sigma_{X}^{2}\;\right)\;\;\left\{\;d_{S,N}-\;\left(\;y+\frac{b}{1-p_{S}}\;\right)\;\;\right\},$$

where*d*_*S*,*N*_denotes the observed value for *D*_*S*,*N*_. We are seeking *f*(*u*|*y*,*z*_*S*_,*Q*_*S*_), which is given by

(A.2) $$\begin{array}{cc} \frac{f\;\left(u,y,z_{S}|Q_{S}\right)}{f\;\left(\;y,z_{S}|Q_{S}\right)} & =\frac{\varphi \left(\frac{z_{S}-\theta_{S}\alpha_{1}}{\sqrt{\sigma_{X}^{2}+\tau_{U}^{2}}}\right)\frac{1}{\tau_{U}^{2}}\varphi \left(\frac{u-z_{S}/\alpha_{1}}{\alpha_{2}}\right)\frac{1}{\sigma_{Y}}\varphi \left(\frac{y-\theta_{S^{c}}}{\sigma_{Y}}\right)1_{Q_{S}}K_{S}^{-1}(\theta )}{\frac{\alpha_{2}}{\tau_{U}^{2}}\varphi \left(\frac{z_{S}-\theta_{S}\alpha_{1}}{\sqrt{\sigma_{X}^{2}+\tau_{U}^{2}}}\right)\frac{1}{\sigma_{Y}}\varphi \left(\frac{y-\theta_{S^{c}}}{\sigma_{Y}}\right)[1-\Phi \{-f_{U}(x,y)\}]1_{Q_{S}}K_{S}^{-1}(\theta )}\\ =\frac{\frac{1}{\alpha_{2}}\varphi \left(\frac{u-z_{S}/\alpha_{1}}{\alpha_{2}}\right)}{1-\Phi \{-f_{U}(x,y)\}}1\left[u<\frac{\tau_{U}}{\sigma_{X}}\left\{z_{S}-\frac{\tau_{U}}{\sigma_{X}}\left(\;y+\frac{b}{1-p_{S}}\right)\right\}\right]. \end{array}$$

We want to obtain the expression for *E*[*U*|*Y*,*Z*_*S*_,*Q*_*S*_] and so we derive $\int \mathrm{uf}\;\left(u|y,z_{S},Q_{S}\right)\mathrm{du}$. Using the standard result that $\overset{a}{\underset{-\infty }{\int }}(r/\sigma )\varphi ((r-\mu )/\sigma )\mathrm{dr}=-\sigma \varphi ((a-\mu )/\sigma )+\mu \Phi ((a-\mu )/\sigma )$ 36 and steps in 15,

$$\int \mathrm{uf}\;\left(u|y,z_{S},Q_{S}\right)\mathrm{du}=\frac{\overset{2}{\underset{U}{\tau }}x+\overset{2}{\underset{X}{\sigma }}u}{\overset{2}{\underset{X}{\sigma }}+\overset{2}{\underset{U}{\tau }}}-\frac{\overset{2}{\underset{U}{\tau }}}{\sqrt{\overset{2}{\underset{X}{\sigma }}+\overset{2}{\underset{U}{\tau }}}}\frac{\varphi \{\;f_{U}(x,y)\}}{\Phi \{\;f_{U}(x,y)\}}=d_{S,N}-\frac{\tau_{U}^{2}}{\sqrt{\sigma_{X}^{2}+\tau_{U}^{2}}}\frac{\varphi \{\;f_{U}(x,y)\}}{\Phi \{\;f_{U}(x,y)\}}$$

so that conditional on continuing to stage 2 with *S*, expression (11) gives the UMVUE for *θ*_*S*_.

## Appendix B Uniformly minimum variance unbiased estimators for *θ*_*S*_ and $\theta_{S^{c}}$ when the full population is selected and *p*_*S*_ is known

### B.1

Here we derive the UMVUEs for *θ*_*S*_ and $\theta_{S^{c}}$ conditional on continuing with *F* in stage 2. We derive the UMVUEs for *θ*_*S*_ and $\theta_{S^{c}}$ separately. Let *Q*_*F*_ denote the event $X\leq Y+b/\left(1-p_{S}\right)$. Conditional on *Q*_*F*_, the density *f*(*v*,*x*,*y*,*w*|*Q*_*F*_) is given by

(B1) $$\frac{1}{\tau_{V}}\varphi \left(\frac{v-\theta_{S}}{\tau_{V}}\right)\frac{1}{\sigma_{X}}\varphi \left(\frac{x-\theta_{S}}{\sigma_{X}}\right)\frac{1}{\sigma_{Y}}\varphi \left(\frac{y-\theta_{S^{c}}}{\sigma_{Y}}\right)\frac{1}{\tau_{W}}\varphi \left(\frac{w-\theta_{S^{c}}}{\tau_{W}}\right)1_{Q_{F}}K_{F}^{-1}(\theta ),$$

where $1_{Q_{F}}$ denotes the indicator function for the event *Q*_*F*_ and *K*_*F*_(*θ*) the probability for event *Q*_*F*_ given the true parameter vector $\theta =(\theta_{S},\theta_{S^{c}})$. The aforementioned density can be re‐expressed as

$$\frac{1}{\tau_{V}}\varphi \left(\frac{\frac{\tau_{V}}{\sigma_{X}}x+\frac{\sigma_{X}}{\tau_{V}}v-\theta_{S}\gamma_{1}}{\sqrt{\sigma_{X}^{2}+\tau_{V}^{2}}}\right)\frac{1}{\sigma_{X}}\varphi \left(\frac{x-\left(\frac{\tau_{V}}{\sigma_{X}}x+\frac{\sigma_{X}}{\tau_{V}}v\right)/\gamma_{1}}{\frac{\sigma_{X}^{2}}{\tau_{V}^{2}}\gamma_{2}}\right)\psi (\;y,w)1_{Q_{F}}K_{F}^{-1}(\theta ),$$

where

$$\gamma_{1}=\frac{\sigma_{X}}{\tau_{V}}+\frac{\tau_{V}}{\sigma_{X}},\;\;\;\gamma_{2}=\frac{\tau_{V}^{2}}{\sqrt{\sigma_{X}^{2}+\tau_{V}^{2}}}\;\;\;\text{and}\;\;\;\psi (\;y,w)=\frac{1}{\sigma_{Y}}\varphi \left(\frac{y-\theta_{S^{c}}}{\sigma_{Y}}\right)\frac{1}{\tau_{W}}\varphi \left(\frac{w-\theta_{S^{c}}}{\tau_{W}}\right).$$

Let $z_{S}^{F}=\left(\tau_{V}/\sigma_{X}\right)x+\left(\sigma_{X}/\tau_{V}\right)v$; from the preceding density, conditional on *Q*_*F*_, $\left(Z_{S}^{F},Y,W\right)$ is sufficient and complete statistic for estimating *θ*_*S*_. Therefore, because conditional on *Q*_*F*_, *V* is an unbiased estimator for *θ*_*S*_, the UMVUE for *θ*_*S*_ is $E\left[V|Z_{S}^{F},Y,W,Q_{F}\right]$. To obtain the expression for $E\left[V|Z_{S}^{F},Y,W,Q_{F}\right]$, we derive the density $f\;\left(v|z_{S}^{F},y,w,Q_{F}\right)=f\;\left(v,z_{S}^{F},y,w|Q_{F}\right)/f\;\left(z_{S}^{F},y,w|Q_{F}\right)$.

The aforementioned density can be transformed to obtain the density $f\;\left(x,z_{S}^{F},y,w|Q_{F}\right)$, which is given by

$$\varphi \left(\frac{z_{S}^{F}-\theta_{S}\gamma_{1}}{\sqrt{\sigma_{X}^{2}+\tau_{V}^{2}}}\right)\frac{1}{\sigma_{X}^{2}}\varphi \left(\frac{x-z_{S}^{F}/\gamma_{1}}{\frac{\sigma_{X}^{2}}{\tau_{V}^{2}}\gamma_{2}}\right)\psi (\;y,w)1_{Q_{F}}K_{F}^{-1}(\theta ),$$

and the density $f\;\left(v,z_{S}^{F},y,w|Q_{F}\right)$, which is given by

$$\varphi \left(\frac{z_{S}^{F}-\theta_{S}\gamma_{1}}{\sqrt{\sigma_{X}^{2}+\tau_{V}^{2}}}\right)\frac{1}{\tau_{V}^{2}}\varphi \left(\frac{v-z_{S}^{F}/\gamma_{1}}{\gamma_{2}}\right)\psi (\;y,w)1_{Q_{F}}K_{F}^{-1}(\theta ).$$

As in Appendix A, the density $f\;\left(z_{S}^{F},y,w|Q_{F}\right)$ is obtained by integrating out *x* in the $f\;\left(x,z_{S}^{F},y,w|Q_{F}\right)$ as follows

$$\begin{array}{cc} f\;\left(z_{S}^{F},y,w|Q_{F}\right) & =\frac{1}{\sigma_{X}^{2}}\varphi \left(\frac{z_{S}^{F}-\theta_{S}\gamma_{1}}{\sqrt{\sigma_{X}^{2}+\tau_{V}^{2}}}\right)\psi (\;y,w)\left\{\overset{y+b/\left(1-p_{S}\right)}{\underset{-\infty }{\int }}\varphi \left(\frac{x-z_{S}^{F}/\gamma_{1}}{\frac{\sigma_{X}^{2}}{\tau_{V}^{2}}\gamma_{2}}\right)\mathrm{dx}\right\}1_{Q_{F}}K_{F}^{-1}(\theta )\\ =\frac{\gamma_{2}}{\tau_{V}^{2}}\varphi \left(\frac{z_{S}^{F}-\theta_{S}\gamma_{1}}{\sqrt{\sigma_{X}^{2}+\tau_{V}^{2}}}\right)\psi (\;y,w)\Phi \{\;f_{V}(x,y)\}1_{Q_{F}}K_{F}^{-1}(\theta ), \end{array}$$

where

$$f_{V}(x,y)=\left(\sqrt{\sigma_{X}^{2}+\tau_{V}^{2}}/\sigma_{X}^{2}\right)\left(\;y+\frac{b}{1-p_{S}}-\frac{\tau_{V}^{2}x+\sigma_{X}^{2}v}{\sqrt{\sigma_{X}^{2}+\tau_{V}^{2}}}\right)=\left(\sqrt{\sigma_{X}^{2}+\tau_{V}^{2}}/\sigma_{X}^{2}\right)\left(\;y+\frac{b}{1-p_{S}}-d_{S,N}^{F}\right),$$

where $d_{S,N}^{F}$ is the observed value for $D_{S,N}^{F}$. We are seeking the density $f\;\left(v|z_{S}^{F},y,w,Q_{F}\right)$

$$\frac{f\;\left(v,z_{S}^{F},y,w|Q_{F}\right)}{f\;\left(z_{S}^{F},y,w|Q_{F}\right)}=\frac{\frac{1}{\gamma_{2}}\varphi \left(\frac{v-z_{S}^{F}/\gamma_{1}}{\gamma_{2}}\right)}{\Phi \{\;f_{V}(x,y)\}}\cdot I\left[v>\frac{\tau_{V}}{\sigma_{X}}\left\{z_{S}^{F}-\frac{\tau_{V}}{\sigma_{X}}\left(\;y+\frac{b}{1-p_{S}}\right)\right\}\right].$$

We want to obtain the expression for $E\left[V|Z_{S}^{F},Y,W,Q_{F}\right]$ and so we derive $\int \mathrm{vf}\;\left(v|\overset{F}{\underset{S}{z}},y,w,Q_{F}\right)\mathrm{dv}$. As in Appendix A, using the standard result that $\overset{a}{\underset{-\infty }{\int }}(r/\sigma )\varphi ((r-\mu )/\sigma )\mathrm{dr}=-\sigma \varphi ((a-\mu )/\sigma )+\mu \Phi ((a-\mu )/\sigma )$ and similar steps as in 15, we can show that

$$\int \mathrm{vf}\;\left(v|\overset{F}{\underset{S}{z}},y,w,Q_{F}\right)\mathrm{dv}=\frac{\overset{2}{\underset{V}{\tau }}x+\overset{2}{\underset{X}{\sigma }}v}{\overset{2}{\underset{X}{\sigma }}+\overset{2}{\underset{V}{\tau }}}+\frac{\overset{2}{\underset{V}{\tau }}}{\sqrt{\overset{2}{\underset{X}{\sigma }}+\overset{2}{\underset{V}{\tau }}}}\frac{\varphi \left\{\;f_{V}(x,y)\right\}}{\Phi \left\{\;f_{V}(x,y)\right\}}=\overset{F}{\underset{S,N}{d}}+\frac{\tau_{V}^{2}}{\sqrt{\sigma_{X}^{2}+\tau_{V}^{2}}}\frac{\varphi \left\{\;f_{V}(x,y)\right\}}{\Phi \left\{\;f_{V}(x,y)\right\}},$$

so that expression (12) gives the UMVUE for *θ*_*S*_ conditional on continuing with *F*.

To obtain the unbiased estimator for $\theta_{S^{c}}$, we note that the density given by expression (B1) can be re‐expressed as

$$\frac{1}{\tau_{W}}\varphi \left(\frac{\frac{\tau_{W}}{\sigma_{Y}}y+\frac{\sigma_{Y}}{\tau_{W}}w-\theta_{S^{c}}\beta_{1}}{\sqrt{\sigma_{Y}^{2}+\tau_{W}^{2}}}\right)\frac{1}{\sigma_{Y}}\varphi \left(\frac{y-\left(\frac{\tau_{W}}{\sigma_{Y}}y+\frac{\sigma_{Y}}{\tau_{2}}w\right)/\beta_{1}}{\frac{\sigma_{Y}^{2}}{\tau_{W}^{2}}\beta_{2}}\right)\psi (x,v)1_{Q_{F}}K_{F}^{-1}(\theta ),$$

where

$$\beta_{1}=\frac{\sigma_{Y}}{\tau_{W}}+\frac{\tau_{W}}{\sigma_{Y}},\;\;\;\beta_{2}=\frac{\tau_{W}^{2}}{\sqrt{\sigma_{Y}^{2}+\tau_{W}^{2}}}\;\;\;\text{and}\;\;\;\psi (x,v)=\frac{1}{\sigma_{X}}\varphi \left(\frac{x-\theta_{S}}{\sigma_{X}}\right)\frac{1}{\tau_{V}}\varphi \left(\frac{v-\theta_{S}}{\tau_{V}}\right).$$

Let $z_{S^{c}}^{F}=\left(\tau_{W}/\sigma_{Y}\right)y+\left(\sigma_{Y}/\tau_{W}\right)w$; from the preceding density, conditional on *Q*_*F*_, $\left(Z_{S^{c}}^{F},X,V\right)$ is sufficient and complete statistic for estimating $\theta_{S^{c}}$. Therefore, because conditional on *Q*_*F*_, *W* is an unbiased estimator for $\theta_{S^{c}}$, the UMVUE for $\theta_{S^{c}}$ is $E\left[W|Z_{S^{c}}^{F},X,V,Q_{F}\right]$. To obtain the expression for $E\left[W|Z_{S^{c}}^{F},X,V,Q_{F}\right]$, we derive the density $f\;\left(w|z_{S^{c}}^{F},x,v,Q_{F}\right)=f\;\left(w,z_{S^{c}}^{F},x,v|Q_{F}\right)/f\;\left(z_{S^{c}}^{F},x,v|Q_{F}\right)$. The aforementioned density can be transformed to obtain the density $f\;\left(\;y,z_{S^{c}}^{F},x,v|Q_{F}\right)$, which is given by

$$\varphi \left(\frac{z_{S^{c}}^{F}-\theta_{S^{c}}\beta_{1}}{\sqrt{\sigma_{Y}^{2}+\tau_{W}^{2}}}\right)\frac{1}{\sigma_{Y}^{2}}\varphi \left(\frac{y-z_{S^{c}}^{F}/\beta_{1}}{\frac{\sigma_{Y}^{2}}{\tau_{W}^{2}}\beta_{2}}\right)\psi (x,v)1_{Q_{F}}K_{F}^{-1}(\theta ),$$

and to obtain the density $f\;\left(w,z_{S^{c}}^{F},x,v|Q_{F}\right)$, which is given by

$$\varphi \left(\frac{z_{S^{c}}^{F}-\theta_{S^{c}}\beta_{1}}{\sqrt{\sigma_{Y}^{2}+\tau_{W}^{2}}}\right)\frac{1}{\tau_{W}^{2}}\varphi \left(\frac{w-z_{S^{c}}^{F}/\beta_{1}}{\beta_{2}}\right)\psi (x,v)1_{Q_{F}}K_{F}^{-1}(\theta ).$$

The density $f\;\left(z_{S^{c}}^{F},x,v|Q_{F}\right)$ is obtained by integrating out *y* as follows

$$\begin{array}{cc} f\;\left(z_{S^{c}}^{F},x,v|Q_{F}\right) & =\frac{1}{\sigma_{Y}^{2}}\varphi \left(\frac{z_{S^{c}}^{F}-\theta_{S^{c}}\beta_{1}}{\sqrt{\sigma_{Y}^{2}+\tau_{W}^{2}}}\right)\psi (x,v)\left\{\overset{\infty }{\underset{x-b/\left(1-p_{S}\right)}{\int }}\varphi \left(\frac{y-z_{S^{c}}^{F}/\beta_{1}}{\frac{\sigma_{Y}^{2}}{\tau_{W}^{2}}\beta_{2}}\right)\mathrm{dy}\right\}1_{Q_{F}}K_{F}^{-1}(\theta )\\ =\frac{\beta_{2}}{\tau_{W}^{2}}\varphi \left(\frac{z_{S^{c}}^{F}-\theta_{S^{c}}\beta_{1}}{\sqrt{\sigma_{Y}^{2}+\tau_{W}^{2}}}\right)\psi (x,v)\left[1-\Phi \left\{-f_{W}(x,y)\right\}\right]1_{Q_{F}}K_{F}^{-1}(\theta ), \end{array}$$

where

$$\begin{array}{c} f_{W}(x,y)=\left(\frac{\sqrt{\sigma_{Y}^{2}+\tau_{W}^{2}}}{\sigma_{Y}^{2}}\right)\left\{\frac{\tau_{W}^{2}y+\sigma_{Y}^{2}w}{\sigma_{Y}^{2}+\tau_{W}^{2}}-\left(x-\frac{b}{1-p_{S}}\right)\right\}=\left(\frac{\sqrt{\sigma_{Y}^{2}+\tau_{W}^{2}}}{\sigma_{Y}^{2}}\right)\left\{d_{S^{c},N}^{F}-\left(x-\frac{b}{1-p_{S}}\right)\right\}, \end{array}$$

where $d_{S^{c},N}^{F}$ is the observed value for $D_{S^{c},N}^{F}$. We are seeking $f\;\left(w|z_{S^{c}}^{F},x,v,Q_{F}\right)$ which, following previous derivation, is given by

$$\begin{array}{c} \frac{f\;\left(w,z_{S^{c}}^{F},x,v|Q_{F}\right)}{f\;\left(z_{S^{c}}^{F},x,v|Q_{F}\right)}=\frac{\frac{1}{\beta_{2}}\varphi \left(\frac{w-z_{S^{c}}^{F}/\beta_{1}}{\beta_{2}}\right)}{1-\Phi \left\{-f_{W}(x,y)\right\}}\cdot I\left[w<\frac{\tau_{W}}{\sigma_{Y}}\left\{z_{S^{c}}^{F}-\frac{\tau_{W}}{\sigma_{Y}}\left(x-\frac{b}{1-p_{S}}\right)\right\}\right]. \end{array}$$

We are seeking the expression for $E\left[W|Z_{S^{c}}^{F},X,V,Q_{F}\right]$ and so we need to obtain $\int \mathrm{wf}\;\left(w|\overset{F}{\underset{S^{c}}{z}},x,v,Q_{F}\right)\mathrm{dw}$. Obtaining this is similar to obtaining $\int \mathrm{uf}\;\left(u|y,z_{S},Q_{S}\right)\mathrm{du}$ in Appendix A and so

$$\begin{array}{c} \int \mathrm{wf}\;\left(w|\overset{F}{\underset{S^{c}}{z}},x,v,Q_{F}\right)\mathrm{dw}=\frac{\overset{2}{\underset{W}{\tau }}y+\overset{2}{\underset{Y}{\sigma }}w}{\overset{2}{\underset{Y}{\sigma }}+\overset{2}{\underset{W}{\tau }}}-\frac{\overset{2}{\underset{W}{\tau }}}{\sqrt{\overset{2}{\underset{Y}{\sigma }}+\overset{2}{\underset{W}{\tau }}}}\frac{\varphi \left\{\;f_{W}(x,y)\right\}}{\Phi \left\{\;f_{W}(x,y)\right\}}=\overset{F}{\underset{S^{c},N}{d}}-\frac{\tau_{W}^{2}}{\sqrt{\sigma_{Y}^{2}+\tau_{W}^{2}}}\frac{\varphi \left\{\;f_{W}(x,y)\right\}}{\Phi \left\{\;f_{W}(x,y)\right\}}, \end{array}$$

so that expression (13) gives the UMVUE for $\theta_{S^{c}}$ conditional on continuing with *F* in stage 2.

## Appendix C Uniformly minimum variance unbiased estimator for *θ*_*S*_ when the subpopulation is selected and *p*_S_ is unknown

### C.1

Here, we derive the UMVUE for *θ*_*S*_ when *S* is selected and *p*_*S*_ is unknown. The derivation is similar to that in Appendix A, and so we will skip some steps. Let $Q_{S}^{*}$ denote the event that *S*_*X*_=*s*_*X*_ and that $X>Y+b/\left(1-{\hat{p}}_{S}\right)$. Conditional on $Q_{S}^{*}$, the density for *f*(*u*,*x*,*y*) is given by

$$\begin{array}{c} \frac{1}{\tau_{U}}\varphi \left(\frac{u-\theta_{S}}{\tau_{U}}\right)\frac{1}{\sigma_{X}}\varphi \left(\frac{x-\theta_{S}}{\sigma_{X}}\right)\frac{1}{\sigma_{Y}}\varphi \left(\frac{y-\theta_{S^{c}}}{\sigma_{Y}}\right)1_{Q_{S}^{*}}K_{S}^{-1}\left(\theta ,p_{S}\right), \end{array}$$

where $1_{Q_{S}^{*}}$ denotes the indicator function for the event $Q_{S}^{*}$, $\theta =\left(\theta ,\theta_{S^{c}}\right)$ and *K*_*S*_(*θ*,*p*_*S*_) the probability for event $Q_{S}^{*}$ given the true parameter vector (*θ*,*p*_*S*_). The density *f*(*u*,*x*,*y*) can be re‐expressed to

$$\begin{array}{c} \frac{1}{\tau_{U}}\varphi \left(\frac{\frac{\tau_{U}}{\sigma_{X}}x+\frac{\sigma_{X}}{\tau_{U}}u-\theta_{S}\alpha_{1}}{\sqrt{\sigma_{X}^{2}+\tau_{U}^{2}}}\right)\frac{1}{\sigma_{X}}\varphi \left(\frac{x-\left(\frac{\tau_{U}}{\sigma_{X}}x+\frac{\sigma_{X}}{\tau_{U}}u\right)/\alpha_{1}}{\frac{\sigma_{X}^{2}}{\tau_{U}^{2}}\alpha_{2}}\right)\frac{1}{\sigma_{Y}}\varphi \left(\frac{y-\theta_{S^{c}}}{\sigma_{Y}}\right)1_{Q_{S}^{*}}K_{S}^{-1}\left(\theta ,p_{S}\right), \end{array}$$

where *α*_1_ and *α*_2_ are as defined in Appendix A. Let *z*_*s*_=(*τ*_*U*_/*σ*_*X*_)*x* + (*σ*_*X*_/*τ*_*U*_)*u*; from the preceding density, conditional on $Q_{S}^{*}$, (*Y*,*Z*_*S*_) is sufficient and complete statistic for estimating *θ*_*S*_. Therefore, because conditional on $Q_{S}^{*}$, *U* is an unbiased estimator for *θ*_*S*_, the UMVUE for *θ*_*S*_ is $E\left[U|Y,Z_{S},Q_{S}^{*}\right]$. To obtain the expression for $E\left[U|Y,Z_{S},Q_{S}^{*}\right]$, we derive the density $f\;\left(u|y,z_{S},Q_{S}^{*}\right)=f\;\left(u,y,z_{S}|Q_{S}^{*}\right)/f\;\left(\;y,z_{S}|Q_{S}^{*}\right)$.

The aforementioned density can be transformed to obtain the density $f\;\left(x,y,z_{S}|Q_{S}^{*}\right)$ given by

$$\begin{array}{c} \varphi \left(\frac{z_{S}-\theta_{S}\alpha_{1}}{\sqrt{\sigma_{X}^{2}+\tau_{U}^{2}}}\right)\frac{1}{\sigma_{X}^{2}}\varphi \left(\frac{x-z_{S}/\alpha_{1}}{\frac{\sigma_{X}^{2}}{\tau_{U}^{2}}\alpha_{2}}\right)\frac{1}{\sigma_{Y}}\varphi \left(\frac{y-\theta_{S^{c}}}{\sigma_{Y}}\right)1_{Q_{S}^{*}}K_{S}^{-1}\left(\theta ,p_{S}\right), \end{array}$$

and to obtain the density $f\;\left(u,y,z_{S}|Q_{S}^{*}\right)$ given by

$$\begin{array}{c} \varphi \left(\frac{z_{S}-\theta_{S}\alpha_{1}}{\sqrt{\sigma_{X}^{2}+\tau_{U}^{2}}}\right)\frac{1}{\tau_{U}^{2}}\varphi \left(\frac{u-z_{S}/\alpha_{1}}{\alpha_{2}}\right)\frac{1}{\sigma_{Y}}\varphi \left(\frac{y-\theta_{S^{c}}}{\sigma_{Y}}\right)1_{Q_{S}^{*}}K_{S}^{-1}\left(\theta ,p_{S}\right). \end{array}$$

Similar to the integration performed to obtain the density given by expression (A.1), the density $f\;\left(\;y,z_{S}|Q_{S}^{*}\right)$ is obtained from $f\;\left(x,y,z_{S}|Q_{S}^{*}\right)$ by integrating out *x* to obtain

$$\begin{array}{c} f\;\left(\;y,z_{S}|Q_{S}^{*}\right)=\frac{\alpha_{2}}{\tau_{U}^{2}}\varphi \left(\frac{z_{S}-\theta_{S}\alpha_{1}}{\sqrt{\sigma_{X}^{2}+\tau_{U}^{2}}}\right)\frac{1}{\sigma_{Y}}\varphi \left(\frac{y-\theta_{S^{c}}}{\sigma_{Y}}\right)[1-\Phi \{-f_{U}(x,y)\}]1_{Q_{S}^{*}}K_{S}^{-1}\left(\theta ,p_{S}\right), \end{array}$$

where $f_{U}(x,y)=\left(\sqrt{\sigma_{X}^{2}+\tau_{U}^{2}}/\sigma_{X}^{2}\right)\left\{d_{S,N}-\left(\;y+\frac{b}{1-s_{X}/n_{1}}\right)\right\}$ and *d*_*S*,*N*_ is the observed value for *D*_*S*,*N*_. We are seeking $f\;\left(u|y,z_{S},Q_{S}^{*}\right)$. Using the property that **1**_*A* ∩ *B*_=**1**_*A*_ **1**_*B*_ and noting that $1_{Q_{S}^{*}}$ cannot be non‐zero if $1_{\left[S_{X}=s_{X}\right]}(\cdot )=0$ and following the steps used to obtain the density given by expression (A.2), then it can be shown that $f\;\left(u|y,z_{S},Q_{S}^{*}\right)$ is given by

$$\begin{array}{c} \frac{f\;\left(u,y,z_{S}|Q_{S}^{*}\right)}{f\;\left(\;y,z_{S}|Q_{S}^{*}\right)}=\frac{\frac{1}{\alpha_{2}}\varphi \left(\frac{u-z_{S}/\alpha_{1}}{\alpha_{2}}\right)}{1-\Phi \{-f_{U}(x,y)\}}1\left[u<\frac{\tau_{U}}{\sigma_{X}}\left\{z_{S}-\frac{\tau_{U}}{\sigma_{X}}\left(\;y+\frac{b}{1-{\hat{p}}_{S}}\right)\right\}\right]. \end{array}$$

The aim is to obtain the expression for $E\left[U|Y,U,Q_{S}^{*}\right]$, which requires derivation of $\int \mathrm{uf}\;\left(u|y,z_{S},Q_{S}^{*}\right)\mathrm{du}$. This can be carried out similar to how $\int \mathrm{uf}\;\left(u|y,z_{S},Q_{S}\right)\mathrm{du}$ is derived in Appendix A, which leads to expression (11) being a UMVUE for *θ*_*S*_ when *p*_*S*_ is unknown and *S* is selected to continue to stage 2.

## Appendix D Uniformly minimum variance unbiased estimators for *θ*_*S*_ and $\theta_{S^{c}}$ when the full population is selected and *p*_*S*_ is unknown

### D.1

Here we derive the UMVUEs for *θ*_*S*_ and $\theta_{S^{c}}$ conditional on continuing with *F* in stage 2 for the case where *p*_*S*_ is unknown. The derivations are similar to those in Appendix B, and so we will skip several steps. Let $Q_{F}^{*}$ denote the event that *S*_*X*_=*s*_*X*_, $X\leq Y+b/\left(1-{\hat{p}}_{S}\right)$ and *S*_*V*_=*s*_*V*_. Conditional on $Q_{F}^{*}$, the density $f\;\left(v,x,y,w|Q_{F}^{*}\right)$ is given by

(D1) $$\frac{1}{\tau_{V}}\varphi \left(\frac{v-\theta_{S}}{\tau_{V}}\right)\frac{1}{\sigma_{X}}\varphi \left(\frac{x-\theta_{S}}{\sigma_{X}}\right)\frac{1}{\sigma_{Y}}\varphi \left(\frac{y-\theta_{S^{c}}}{\sigma_{Y}}\right)\frac{1}{\tau_{W}}\varphi \left(\frac{w-\theta_{S^{c}}}{\tau_{W}}\right)1_{Q_{F}^{*}}K_{F}^{-1}\left(\theta ,p_{S}\right),$$

where $1_{Q_{F}^{*}}$ denotes the indicator function for the event $Q_{F}^{*}$, $\theta =\left(\theta_{S},\theta_{S^{c}}\right)$ and *K*_*F*_(*θ*,*p*_*S*_) the probability for event $Q_{F}^{*}$ given the true parameter vector (*θ*,*p*_*S*_). The aforementioned density can be re‐expressed as

$$\begin{array}{c} \frac{1}{\tau_{V}}\varphi \left(\frac{\frac{\tau_{V}}{\sigma_{X}}x+\frac{\sigma_{X}}{\tau_{V}}v-\theta_{S}\gamma_{1}}{\sqrt{\sigma_{X}^{2}+\tau_{V}^{2}}}\right)\frac{1}{\sigma_{X}}\varphi \left(\frac{x-\left(\frac{\tau_{V}}{\sigma_{X}}x+\frac{\sigma_{X}}{\tau_{V}}v\right)/\gamma_{1}}{\frac{\sigma_{X}^{2}}{\tau_{V}^{2}}\gamma_{2}}\right)\psi (\;y,w)1_{Q_{F}^{*}}K_{F}^{-1}\left(\theta ,p_{S}\right), \end{array}$$

where *γ*_1_, *γ*_2_ and *ψ*(*y*,*w*) are as defined in Appendix B. Let $z_{S}^{F}=\left(\tau_{V}/\sigma_{X}\right)x+\left(\sigma_{X}/\tau_{V}\right)v$; from the preceding density, conditional on $Q_{F}^{*}$, $\left(Z_{S}^{F},Y,W\right)$ is sufficient and complete statistic for estimating *θ*_*S*_. Therefore, because conditional on $Q_{F}^{*}$, *V* is an unbiased estimator for *θ*_*S*_, the UMVUE for *θ*_*S*_ is $E\left[V|Z_{S}^{F},Y,W,Q_{F}^{*}\right]$. To obtain the expression for $E\left[V|Z_{S}^{F},Y,W,Q_{F}^{*}\right]$, we derive the density $f\;\left(v|z_{S}^{F},y,w,Q_{F}^{*}\right)=f\;\left(v,z_{S}^{F},y,w|Q_{F}^{*}\right)/f\;\left(z_{S}^{F},y,w|Q_{F}^{*}\right)$.

The aforementioned density can be transformed to obtain the density $f\;\left(x,z_{S}^{F},y,w|Q_{F}^{*}\right)$, which is given by

$$\begin{array}{c} \varphi \left(\frac{z_{S}^{F}-\theta_{S}\gamma_{1}}{\sqrt{\sigma_{X}^{2}+\tau_{V}^{2}}}\right)\frac{1}{\sigma_{X}^{2}}\varphi \left(\frac{x-z_{S}^{F}/\gamma_{1}}{\frac{\sigma_{X}^{2}}{\tau_{V}^{2}}\gamma_{2}}\right)\psi (\;y,w)1_{Q_{F}^{*}}K_{F}^{-1}\left(\theta ,p_{S}\right) \end{array}$$

and the density $f\;\left(v,z_{S}^{F},y,w|Q_{F}^{*}\right)$, which is given by

$$\begin{array}{c} \varphi \left(\frac{z_{S}^{F}-\theta_{S}\gamma_{1}}{\sqrt{\sigma_{X}^{2}+\tau_{V}^{2}}}\right)\frac{1}{\tau_{V}^{2}}\varphi \left(\frac{v-z_{S}^{F}/\gamma_{1}}{\gamma_{2}}\right)\psi (\;y,w)1_{Q_{F}^{*}}K_{F}^{-1}\left(\theta ,p_{S}\right). \end{array}$$

Similar to integration performed to obtain the density given by expression (A.1), the density $f\;\left(z_{S}^{F},y,w|Q_{F}^{*}\right)$ is obtained by integrating out *x* in $f\;\left(x,z_{S}^{F},y,w|Q_{F}^{*}\right)$ to obtain

$$\begin{array}{c} f\;\left(z_{S}^{F},y,w|Q_{F}^{*}\right)=\frac{\gamma_{2}}{\tau_{V}^{2}}\varphi \left(\frac{z_{S}^{F}-\theta_{S}\gamma_{1}}{\sqrt{\sigma_{X}^{2}+\tau_{V}^{2}}}\right)\psi (\;y,w)\Phi \{\;f_{V}(x,y)\}1_{Q_{F}^{*}}K_{F}^{-1}\left(\theta ,p_{S}\right), \end{array}$$

where $f_{V}(x,y)=\left(\sqrt{\sigma_{X}^{2}+\tau_{V}^{2}}/\sigma_{X}^{2}\right)\left(\;y+\frac{b}{1-s_{X}/n_{1}}-d_{S,N}^{F}\right)$ and $d_{S,N}^{F}$ is the observed value for $D_{S,N}^{F}$. We are seeking the density $f\;\left(v|z_{S}^{F},y,w,Q_{F}^{*}\right)$. Using the property that **1**_*A* ∩ *B*_=**1**_*A*_ **1**_*B*_ and noting that $1_{Q_{F}^{*}}$ cannot be non‐zero if $1_{\left[S_{X}=s_{X}\right]}(\cdot )=0$ or $1_{\left[S_{V}=s_{V}\right]}(\cdot )=0$ and following steps used to obtain the density given by expression (A.2), then it can be shown that $f\;\left(v|z_{S}^{F},y,w,Q_{F}^{*}\right)$ is given by

$$\begin{array}{c} \frac{f\;\left(v,z_{S}^{F},y,w|Q_{F}^{*}\right)}{f\;\left(z_{S}^{F},y,w|Q_{F}^{*}\right)}=\frac{\frac{1}{\gamma_{2}}\varphi \left(\frac{v-z_{S}^{F}/\gamma_{1}}{\gamma_{2}}\right)}{\Phi \{\;f_{V}(x,y)\}}\cdot I\left[v>\frac{\tau_{V}}{\sigma_{X}}\left\{z_{S}^{F}-\frac{\tau_{V}}{\sigma_{X}}\left(\;y+\frac{b}{1-{\hat{p}}_{S}}\right)\right\}\right]. \end{array}$$

We want to obtain the expression for $E\left[V|Z_{S}^{F},Y,W,Q_{F}^{*}\right]$, which requires derivation of $\int \mathrm{vf}\;\left(v|\overset{F}{\underset{S}{z}},y,\right.$ $\left.w,Q_{F}^{*}\right)\mathrm{dv}$. This can be carried out similar to how $\int \mathrm{vf}\;\left(v|\overset{F}{\underset{S}{z}},y,w,Q_{F}\right)\mathrm{dv}$ is derived in Appendix B, which leads to expression (12) giving a UMVUE for *θ*_*S*_ when *p*_*S*_ is unknown and *F* is selected to continue to stage 2.

Combining the derivation for the UMVUE for $\theta_{S^{c}}$ when *p*_*S*_ is known and derivation for the UMVUE for *θ*_*S*_ when *p*_*S*_ is unknown, one can show that expression (13) is a UMVUE for $\theta_{S^{c}}$ when *p*_*S*_ is unknown and *F* is selected to continue to stage 2.
